# Ubiquitin and Ubiquitin-Like Proteins in the Critical Equilibrium between Synapse Physiology and Intellectual Disability

**DOI:** 10.1523/ENEURO.0137-20.2020

**Published:** 2020-08-21

**Authors:** Alessandra Folci, Filippo Mirabella, Matteo Fossati

**Affiliations:** 1 Humanitas Clinical and Research Center-IRCCS, via Manzoni 56, 20089, Rozzano (MI), Italy; 2 Department of Biomedical Sciences, Humanitas University, Via Rita Levi Montalcini 4, 20090 Pieve 9 Emanuele – Milan, Italy; 3 CNR–Institute of Neuroscience, via Manzoni 56, 20089, Rozzano (MI), Italy

**Keywords:** intellectual disability, ubiquitination, sumoylation, neddylation, synapse development, synapse function

## Abstract

Posttranslational modifications (PTMs) represent a dynamic regulatory system that precisely modulates the functional organization of synapses. PTMs consist in target modifications by small chemical moieties or conjugation of lipids, sugars or polypeptides. Among them, ubiquitin and a large family of ubiquitin-like proteins (UBLs) share several features such as the structure of the small protein modifiers, the enzymatic cascades mediating the conjugation process, and the targeted aminoacidic residue. In the brain, ubiquitination and two UBLs, namely sumoylation and the recently discovered neddylation orchestrate fundamental processes including synapse formation, maturation and plasticity, and their alteration is thought to contribute to the development of neurological disorders. Remarkably, emerging evidence suggests that these pathways tightly interplay to modulate the function of several proteins that possess pivotal roles for brain homeostasis as well as failure of this crosstalk seems to be implicated in the development of brain pathologies. In this review, we outline the role of ubiquitination, sumoylation, neddylation, and their functional interplay in synapse physiology and discuss their implication in the molecular pathogenesis of intellectual disability (ID), a neurodevelopmental disorder that is frequently comorbid with a wide spectrum of brain pathologies. Finally, we propose a few outlooks that might contribute to better understand the complexity of these regulatory systems in regard to neuronal circuit pathophysiology.

## Significance Statement

Ubiquitination, sumoylation, and neddylation are related PTMs modulating cellular and molecular pathways that are essential to generate fully functional neuronal circuits. Their impairment is indeed implicated in the pathogenesis of several disorders, including ID. Growing evidence now indicates they also functionally cooperate to govern synapse development and function. The main goals of this review are (1) to provide an overview of the current knowledge on the role of ubiquitination, sumoylation, and neddylation in synapse functions; (2) discuss how altered ubiquitination or sumoylation pathways may contribute to ID development; and (3) highlight evidence of a dynamic cross talk between these PTMs, which represents a novel mechanism that could lead to the identification of new principles underlying synaptic function and dysfunction in ID.

## Introduction

Synapses are the basic functional units of the brain ensuring proper information processing and storage. In the forebrain, glutamate and GABA are the main excitatory and inhibitory neurotransmitters (NTs), respectively. Synaptic transmission occurs when an action potential reaches the active zone of the presynaptic terminal and triggers the Ca^2+^-dependent fusion of synaptic vesicles (SVs) to the presynaptic membrane (Südhof, 2004). SV fusion releases NT molecules into the synaptic cleft and convey a signal to NT receptors localized in the membrane of a specialized domain of the postsynaptic neuron, called the postsynaptic density (PSD). The PSD is composed by a complex network of distinct protein categories. Among them, postsynaptic NT receptors transduce the signal conveyed by NTs into electrical and biochemical cascades. Underneath the postsynaptic membrane, a meshwork of scaffolding proteins generates a structural platform governing synapse organization via multiple bindings to NT receptors, adhesion proteins, signaling molecules, and cytoskeletal elements (Sheng and Kim, 2011).

A prominent feature of synapses is their ability to dynamically modify the strength of synaptic transmission according to the inputs they receive, in a process called synaptic plasticity. Synaptic plasticity is thought to underlie learning and memory and is fundamental to adapt our behavior based on experience. During brain development, synaptic plasticity peaks in specific temporal windows of high sensitivity, called critical periods (Hensch, 2005), which are crucial to orchestrate the formation and refinement of synaptic networks and, ultimately, enables the acquisition of a given skill and/or cognitive function. The drawback of this particularly high sensitivity is a marked susceptibility to genetic and environmental insults that can interfere with molecular and cellular processes critical to synapse development, function, and plasticity. Indeed, perturbation of these pathways leads to neurodevelopmental and psychiatric disorders, such as autism, ID, and schizophrenia. Since these pathologies are characterized by the convergence of distinct pathways onto synaptic impairment, they are collectively defined as synaptopathies (Van Spronsen and Hoogenraad, 2010). To date, no effective therapies are available to treat these diseases. It is therefore crucial to understand the molecular mechanisms of synaptic dysfunction to provide the rationale to develop innovative therapeutic approaches.

Given the plastic nature of the brain, synapses have developed strategies to rapidly modify the strength of synaptic transmission. The dynamic modulation of protein activity via PTMs is a major mechanism to efficiently tune synapse assembly, maturation, and function. PTMs refer to covalent enzymatic modifications, either reversible or irreversible of target proteins, following their translation (Bürkle, 2001). They typically consist in the addition of a functional moiety, which can be either chemical groups or complex molecules, including lipids, sugars, nucleosides, and polypeptides to specific residues of target proteins. PTMs regulate multiple aspects of protein physiology from subcellular localization and activity to conformation and stability/turnover. In neurons, PTMs have been extensively investigated and modulate virtually all pathways that are required to ensure proper synaptic transmission and plasticity, such as presynaptic NT release (Takahashi et al., 2003; Hegde and DiAntonio, 2002; Schorova and Martin, 2016), trafficking and biophysical properties of NT receptors (Luscher et al., 2011; Schorova and Martin, 2016; Diering and Huganir, 2018), PSD organization (Zacchi et al., 2014; Coba, 2019), and synaptic adhesion (Jeong et al., 2017). Beyond the essential roles of PTMs in brain physiology, their impairment is thought to critically contribute to the etiology of several brain disorders including synaptopathies. Among PTMs, ubiquitination and ubiquitin-like proteins (UBLs) such as sumoylation and neddylation share multiple features, tightly interplay, and are vital to synapse assembly, maturation, and function. In this review, we focus on the role of ubiquitination, sumoylation, and neddylation in synapse physiology and their implication in the molecular pathogenesis of ID, a generalized neurodevelopmental disorder that manifests in a wide range of brain pathologies (Verpelli and Sala, 2012; Picker and Walsh, 2013; Vissers et al., 2016).

## Ubiquitination and UBL Pathways in Synapse Physiology

### Ubiquitination

Ubiquitination occurs in all eukaryotic cells. It consists in the reversible conjugation of the 76 amino acid (aa)-long ubiquitin protein to lysine (K) residues of target proteins. This process is catalyzed by a series of enzymatic reactions. E1 ubiquitin enzymes bind to and activate free ubiquitin through adenylation at ubiquitin C-terminal and thiol transfer. Activated ubiquitin is then transferred to E2 ubiquitin-conjugating enzymes and finally transferred onto a K residue of target proteins by E3 ubiquitin ligases. Protein substrates can be either mono-ubiquitinated or poly-ubiquitinated and ubiquitin chains can vary depending on which of the seven K residues of ubiquitin is used to covalently attach the subsequent ubiquitin (for a comprehensive review, see Komander and Rape, 2012). The human genome encodes two E1, ∼50 E2, and ∼600 E3 enzymes. Thus, substrate specificity mainly relies on E3 ligases and different combinations of E2-E3 proteins. Ubiquitination is counterbalanced by the action of deubiquitinating enzymes (DUBs) that remove ubiquitin from target proteins. The coordinated activity of ubiquitinating enzymes and DUBs is essential to set and maintain ubiquitin homeostasis (Kowalski and Juo, 2012).

Protein degradation through the ubiquitin proteasome system (UPS) is the best characterized function of ubiquitination and K48 poly-ubiquitin chains are the most common signal to target proteins for degradation. Conversely, mono-ubiquitination and K63 poly-ubiquitin chains mediate non-proteasomal functions and typically modulate phosphorylation-dependent protein activation, protein-protein interactions, and membrane protein trafficking (Komander and Rape, 2012). Both proteasomal and non-proteasomal ubiquitin conjugations occur at the synapse, where they regulate molecular processes important for synapse formation, maturation, and plasticity. As a consequence, ubiquitination is critical for long-term memory formation and stability as observed with a variety of behavioral paradigms (for review, see Jarome and Helmstetter, 2013).

#### Presynaptic ubiquitination

**Figure 1.**
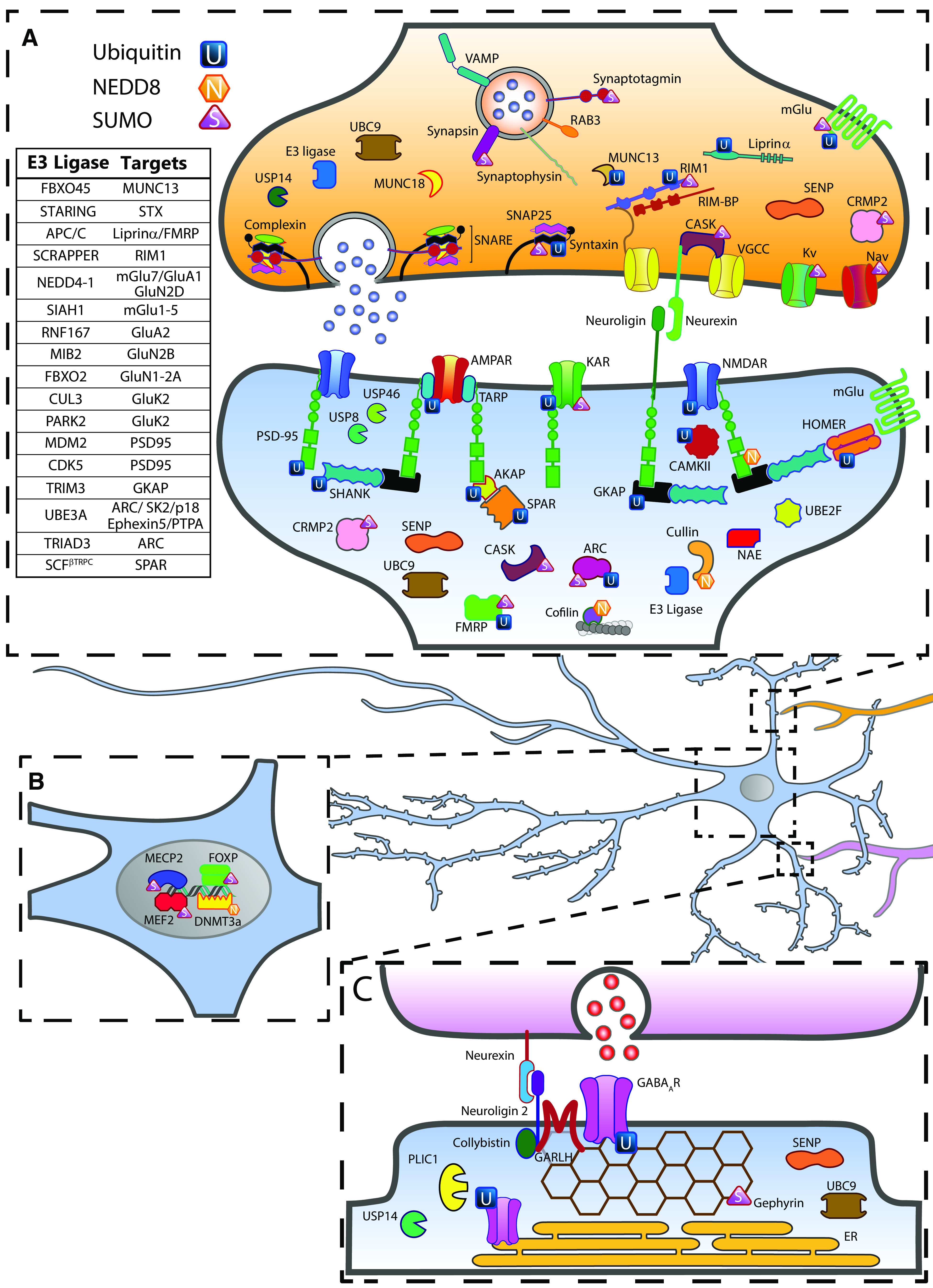
Neuronal ubiquitination and ubiquitin-like modifications. ***A***, ***C***, Major components of excitatory and inhibitory synapses targeted by ubiquitin (blue squares) SUMO (purple triangles) and NEDD8 (orange hexagons) pathways. Deubiquitinating enzymes (green clamshell-like shapes) and components of the SUMO (UBC9 and SENPs) and NEDD8 machineries (NAE, UBC12, and UBE2F) are also indicated. Although NEDD8 pathway and targets are also present in the presynaptic compartment, for simplicity they are depicted in the postsynaptic region only. In ***A***, E3 ubiquitin ligases operating at excitatory synapses and their known substrates are listed in the left table. ***B***, Nuclear sumoylation and neddylation critical to synaptic function are indicated.

The importance of ubiquitination to the presynaptic function was first described in *Drosophila* neuromuscular junctions (NMJs). Pharmacological and genetic perturbations of the UPS lead to the accumulation of Dunc-13, a protein important for SV release, and significantly increase presynaptic efficacy (Speese et al., 2003). In line with this, MUN-13, the mouse ortholog of *Dunc-13* is also ubiquitinated by the E3 ligase FBXO45 in the hippocampus (Tada et al., 2010; Fig. 1*A*). Syntaxin 1 (STX1), a major component of the SNAP REceptor (SNARE) complex and the binding partner of the presynaptic Ca^2+^ sensor synaptotagmin (Rizo and Xu, 2015), is poly-ubiquitinated by the E3 enzyme Staring (Chin et al., 2002; Fig. 1*A*). Ubiquitination further regulates NT release by targeting the presynaptically expressed Group III metabotropic glutamate (mGlu)7 receptor, which inhibits glutamate and GABA release (Niswender and Conn, 2010). Upon stimulation with L-glutamate, the E3 ligase NEDD4 induces rapid mGlu7 internalization and degradation via both proteasomal and lysosomal pathways (Lee et al., 2019; Fig. 1*A*). Protein degradation via the UPS is also a major regulator of SV recycling (Willeumier et al., 2006). Interestingly, blocking action potentials with tetrodotoxin (TTX) prevents the effect of proteasome inhibition on the recycling vesicle pool, suggesting that presynaptic UPS may be a negative feedback-regulator of synaptic transmission.

Beside basal synaptic transmission, presynaptic ubiquitination is also a critical determinant of synaptic plasticity and is required for cognition. Genetic deletion of the gene enconding the anaphase-promoting complex/cyclosome (APC/C) in the forebrain of adult mice impairs hippocampal-dependent memories, such as spatial memory and extinction of fear memory resulting in anxiety-related behaviors (Li et al., 2008; Kuczera et al., 2010). While at *Drosophila* and *Caenorhabditis elegans* NMJs it is known that APC/C targets the active zone protein Liprin-α (van Roessel et al., 2004; Kowalski et al., 2014), the presynaptic targets in mammals remain elusive. Recently, the E3 ligase RNF8 was shown to be required to build up cerebellar circuits mediating procedural motor learning by limiting the formation of parallel fiber presynaptic boutons onto Purkinje cells by targeting as yet unidentified substrates (Valnegri et al., 2017). Another presynaptic E3 ligase fundamental to establish neuronal connectivity is SCRAPPER. In mouse hippocampal neurons, SCRAPPER modulates multiple aspects of the presynaptic function by directly ubiquitinating and regulating Rab3-interacting protein 1 (RIM1; Fig. 1*A*). Consistent with the role of RIM1 as major SV priming factor (Südhof, 2004), axonal boutons of *Scrapper*-knock-out (KO) neurons show enhanced synaptic transmission, owing a significant increase of NT release probability (Yao et al., 2007; Takagi et al., 2012). Moreover, the absence of *Scrapper* expression interferes with performances in contextual fear conditioning tests and hippocampi derived from these mice exhibit bidirectional changes of synaptic plasticity regulation (also referred to as metaplasticity; Yao et al., 2011; Takagi et al., 2012). These cellular and behavioral features partially recapitulate defects observed in *Rim1*-KO animals (Castillo et al., 2002; Powell et al., 2004), although there may be other SCRAPPER synaptic targets. Finally, a spontaneous mutation in the *Usp14* gene, encoding the proteasome-associated deubiquitinating enzyme USP14 (Fig. 1*A*), leads to progressive locomotor defects and ataxia (Wilson et al., 2002). Loss of *Usp14* results in impaired short-term facilitation and reduced SV number, indicating the importance of a balanced ubiquitination to preserve presynaptic functions (Walters et al., 2014). However, which proteins are targeted by USP14 and whether USP14 facilitates or inhibits presynaptic UPS are still unclear.

Intriguingly, two large scaffolding proteins of the active zone, Piccolo and Bassoon, were suggested to be important regulators of presynaptic ubiquitination (Waites et al., 2013). Interference with the expression of these genes leads to aberrant degradation of proteins operating in the active zone and degeneration of presynaptic boutons. As this phenotype is partially rescued by either the inhibition of the proteasome or the downregulation of the E3 ligase SIAH1, it is likely that Piccolo and Bassoon limit presynaptic ubiquitination via the suppression of SIAH1 activity.

Altogether, the aforementioned studies clearly indicate that ubiquitination is critical to NT release, the primary function of presynaptic terminals, and at the circuit level participates to memory and learning processes.

#### Postsynaptic ubiquitination

At the postsynaptic site, ubiquitination is a key mechanism that shapes the functional organization of both excitatory and inhibitory synapses by targeting multiple categories of postsynaptic proteins (Fig. 1*A*,*C*). Consistent with this, the proteasome is rapidly recruited and trapped into dendritic spines, the major site for excitatory synapses, after membrane depolarization (Bingol and Schuman, 2006; Bingol et al., 2010).

**Figure 2.**
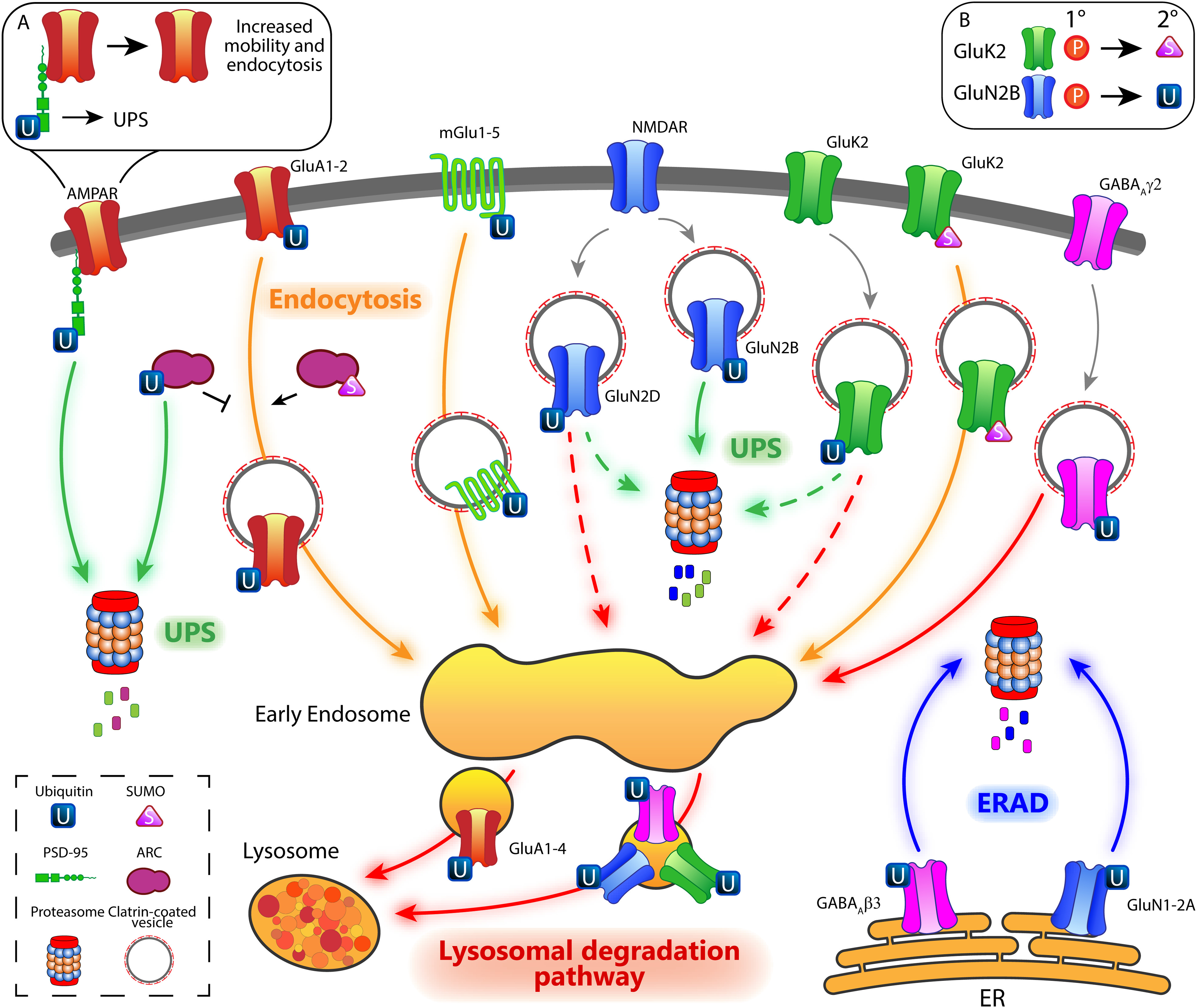
Postsynaptic control of glutamate and GABA_A_ receptors by ubiquitination and sumoylation. UPS-dependent degradation (green arrows) of PSD-95 destabilizes surface AMPARs, resulting in enhanced receptor lateral mobility and consequently, endocytosis (box ***A***). Ubiquitination of GluA1 and GluA2 decreases surface AMPARs through clathrin-dependent endocytosis (orange arrows). Ubiquitinated ARC is degraded via the UPS pathway. As ARC is a major regulator of AMPAR internalization, reduced ARC levels suppress AMPAR endocytosis. Conversely, sumoylated ARC triggers AMPAR internalization. Moreover, ubiquitination of intracellular GluA1-4 may also promote AMPAR sorting to the lysosomal degradation pathway (red arrows). Ubiquitination of mGlu1-5 receptors enhanced their clathrin-dependent endocytosis. Surface NMDARs are regulated by the ubiquitination pathway in a subunit-dependent manner. GluN2B undergoes a phosphorylation-dependent ubiquitination (box ***B***), leading to UPS-dependent degradation of NMDARs. GluN2D ubiquitination enhances its degradation, although it is not clear whether it utilizes UPS-dependent or lysosomal-dependent pathways (green and red dotted arrows). Finally, ubiquitination of newly synthetized GluN1 and GluN2A results in NMDAR retrotranslocation from the ER to the cytosol and subsequent degradation through the ERAD pathway (blue arrows). Similar to GluN2D, GluK2 ubiquitination is phosphorylation-dependent and triggers its degradation through an as yet ill-defined pathway (green and red dotted arrows). In contrast, sumoylated GluK2-containing KARs are removed from the synaptic membrane via clathrin-dependent endocytosis. At inhibitory synapses, ubiquitinated γ2-containing GABA_A_R are sorted to the lysosomal degradation pathway, while β3-containing GABA_A_R are ubiquitinated in the ER and degraded through the ERAD machinery.

One of the most well-studied ubiquitinated NT receptors are AMPA receptors (AMPARs; Figs. 1*A*, 2). They are glutamate-gated ion channels (also referred to as ionotropic glutamate receptors, iGluRs) and mediate the fast excitatory transmission in the brain (Greger et al., 2017). The regulation of their number at the synaptic surface is a major determinant of synaptic strength and plasticity (Choquet and Triller, 2013; Huganir and Nicoll, 2013). The importance of AMPAR ubiquitination was first demonstrated in *C. elegans*, where it controls synaptic abundance of these receptors and locomotor behavior (Burbea et al., 2002; Juo and Kaplan, 2004; Dreier et al., 2005; Emtage et al., 2009). In mammals, the selective ubiquitination of GluA1 and GluA2 subunits is required for the activity-dependent endocytosis of surface AMPARs directing them toward degradative pathways. Ubiquitination occurs at K residues of their intracellular C-terminal tail via the E3 ligases NEDD4-1 and RNF167 (Schwarz et al., 2010; Lussier et al., 2011, 2012). A more recent study showed that activity-dependent AMPAR ubiquitination involves all four GluA subunits (GluA1-4) and sorts AMPARs from early to late endosomes and subsequent lysosome-dependent degradation (Widagdo et al., 2015). Considering that the modulation of AMPAR number at synaptic sites tunes synaptic strength and plasticity, it is expected that AMPAR ubiquitination has implication in learning and memory paradigms. Consistently, proteasome inhibition increases synaptic surface GluA1-containing AMPARs and enhances fear-conditioned learning (Yeh et al., 2006). UPS-dependent degradation of GluA1 also facilitates the extinction of fear memories, which is mediated by NMDAR-dependent depression of synaptic activity (Mao et al., 2008). AMPAR ubiquitination is finely counteracted by the opposite action of a few DUBs. Overexpression of *Usp46* prolongs GluA1 half-life, resulting in enhanced amplitude of excitatory postsynaptic currents (Huo et al., 2015). USP8 antagonizes the E3 ligase NEDD4-1 and is involved in downscaling synaptic strength during homeostatic plasticity, providing the first evidence for an opposed bidirectional control of synaptic strength by ubiquitination (Scudder et al., 2014).

Among the iGluR family, NMDA receptors (NMDARs) play an essential role in synaptic plasticity (Paoletti et al., 2013), and their function, similarly to other iGluR members, is modulated by ubiquitination (Figs. 1*A*, 2). The GluN2B, GluN2D, GluN1, and GluN2A subunits are targeted by the E3 ubiquitin ligases Mind bomb-2 (MIB2), NEDD4, and FBXO2 (Kato et al., 2005; Jurd et al., 2008; Gautam et al., 2013; Atkin et al., 2015). Upon phosphorylation-dependent ubiquitination of GluN2B by MIB2 in the PSD, NMDAR-mediated currents are significantly reduced. This effect is prevented when cells are treated with the proteasome inhibitor MG132, suggesting that ubiquitinated GluN2B undergoes UPS-dependent degradation (Jurd et al., 2008). Similarly, NEDD4 selectively conjugates poly-ubiquitin chains to GluN2D and reduces NMDAR currents in heterologous cells (Gautam et al., 2013). Whether ubiquitination directly mediates GluN2D proteasome-dependent degradation or modulates receptor endocytosis and sorting to late endosomes and lysosomes is not known. Conversely, FBXO2 ubiquitinates newly synthetized GluN1 and GluN2A subunits in the endoplasmic reticulum (ER) and mediates its degradation via the ER-associated degradation (ERAD) machinery, a mechanism that avoids the formation of supra-numerary synapses in the dendritic shaft and aberrant NMDAR-mediated currents (Kato et al., 2005; Nelson et al., 2006; Gascón et al., 2007; Atkin et al., 2015). Synaptic abundance and clustering of GluN1-containing NMDARs are also modulated by the hominoid-specific DUB USP6 (Zeng et al., 2019). By generating a humanized knock-in (KI) mouse expressing USP6 under the control of Ca^2+^/calmodulin-dependent kinase II (*CamkII*) promoter, the authors found that USP6 stabilizes NMDARs at synapses and enhances synaptic function resulting in improved mouse cognitive abilities. Beside the novel synaptic mechanism uncovered here, this study suggests for the first time that ubiquitination may have contributed to the evolution of human-specific synaptic features, which are thought to form, together with other processes the cellular basis of human intelligence (DeFelipe, 2011; Geschwind and Rakic, 2013). In line with this, perturbation of USP6 is associated with human-specific neuropsychiatric disorders, such as ID (Ou et al., 2006) and autism spectrum disorders (ASD; Tentler et al., 2003).

Kainate receptors (KARs) are the third receptor subtypes of iGluRs whose activity and trafficking are modulated by ubiquitination (Figs. 1*A*, 2). The E3 ligases Cullin 3 (CUL3) and Parkin 2 (PARK2) target the GluK2 subunit of KARs and regulate its surface expression (Salinas et al., 2006; Maraschi et al., 2014). Strikingly, loss-of-function of PARK2, which is causative of the most common form of familial juvenile parkinsonism, leads to abnormal levels of synaptic GluK2, a mechanism that could underlie glutamate excitotoxicity and neurodegeneration in Parkinson’s disease. Yet, it is unclear whether KAR ubiquitination occurs on surface receptors and whether it is degraded through UPS or lysosomal pathway.

mGlu receptors are also targeted by ubiquitination at the postsynaptic compartment (Figs. 1*A*, 2). Upon dihydroxyphenylglycine (DHPG)-induced activation of Group I mGlu1-5, the E3 ligase SIAH1 attaches K63-linked poly-ubiquitin chains to intracellular K residues of these receptors and induces their internalization (Moriyoshi et al., 2004; Ko et al., 2012; Gulia et al., 2017). Since their activation is critical for the expression of long-term depression (LTD; Niswender and Conn, 2010), mGlu receptor ubiquitination emerged as an essential regulatory mechanism limiting excessive synaptic depression (Gulia et al., 2017).

As aforementioned, postsynaptic scaffolding proteins are the structural core of the PSD. Here, complex protein-protein interactions are tightly regulated by PTMs to control the functional organization of synapses (Sheng and Hoogenraad, 2007). Given the multiple protein-protein interactions that each scaffolding protein engages, the ubiquitination of a few scaffolding proteins allows the precise regulation of a large set of postsynaptic molecules (Fig. 1*A*). PSD-95, GKAP, AKAP79/150, SHANK, and HOMER1A are abundant scaffolding proteins of excitatory synapses and their levels are bidirectionally modulated by ubiquitination in an activity-dependent manner (Ageta et al., 2001; Colledge et al., 2003; Ehlers, 2003; Rezvani et al., 2007; Na et al., 2012). Importantly, the targeted degradation of scaffolding molecules critically contributes to learning and behavior. Retrieval of fear memory upregulates polyubiquitinated SHANK and GKAP, but not PSD-95. Consistent with this, infusion of the proteasome inhibitor clasto-lactacystin-β-lactone in the CA1 region of the hippocampus immediately after retrieval prevents extinction of fear memory (Lee et al., 2008). To date, only few of the E3 ligases targeting scaffolding proteins were identified. The E3 ligase MDM2 ubiquitinates PSD-95 at multiple residues and induces distinct pathways. The mono-ubiquitination of PSD-95 within the PEST (peptides rich in proline, glutamate, serine, and threonine) motif, a short sequence serving as proteolytic signal, triggers PSD-95 degradation. As a consequence, the number of PSD-95 molecules available to anchor AMPARs is reduced, resulting in less stable surface AMPARs and enhanced endocytosis (Fig. 2), a mechanism that critically contributes to hippocampal LTD (Colledge et al., 2003). Conversely, the CDK5-dependent ubiquitination of PSD-95 at K10 promotes its interaction with and the recruitment of the clathrin adaptor protein complex (AP)2 at the synapse, triggering clathrin-dependent AMPAR endocytosis (Bianchetta et al., 2011). The ubiquitination of GKAP, mediated by the E3 ubiquitin ligase TRIM3, induces structural changes of dendritic spines (Hung et al., 2010). While the enzymes mediating SHANK ubiquitination are not known, it has been recently found that USP8 selectively deubiquitinates SHANK1 and SHANK3 and modulates dendritic spine density and morphology (Campbell and Sheng, 2018).

Among postsynaptic signaling proteins, the ubiquitination of the immediate early gene activity-regulated cytoskeleton-associated protein ARC is best characterized (Figs. 1*A*, 2). ARC responds to various forms of synaptic plasticity and promotes the endocytosis of AMPARs (Shepherd et al., 2006; Waung et al., 2008). *UBE3A* is the main causal gene of a severe neurodevelopmental disorder, the Angelman syndrome (AS), and encodes the first E3 ligase proposed to target ARC (Greer et al., 2010). Since UBE3A is also a transcriptional coregulator (Nawaz et al., 1999), it is unclear whether UBE3A-dependent regulation of ARC operates at the protein level through ubiquitination or at the transcriptional level (Kühnle et al., 2013). Other studies indicated that ARC is ubiquitinated by the E3 ligase TRIAD3 (Na et al., 2012; Mabb et al., 2014). Another signaling protein targeted by ubiquitination is the spine-associated Rap GTPase activating protein (SPAR; Fig. 1*A*). SPAR forms a complex with PSD-95 and NMDARs and is critical for spine structural plasticity (Pak et al., 2001). Upon induction of synaptic down-scaling, phosphorylated SPAR is ubiquitinated by the E3 ligase complex SCF^βTRPC^ resulting in its degradation and spine morphology changes (Pak and Sheng, 2003; Ang et al., 2008). Eventually, proteomic approaches aimed at identifying synaptic ubiquitome in rat brains revealed that the CAMKII, which is crucial for the expression of synaptic plasticity (Coultrap and Bayer, 2012), is ubiquitinated (Na et al., 2012; Fig. 1*A*). Yet, the identity of the E3 ligase that targets CAMKII, the consequences on CAMKII stability and the functional relevance for plasticity remain to be assessed.

Ubiquitination was also studied at inhibitory synapses (Fig. 1*C*). The number of surface GABA_A_Rs is critically modulated by the ubiquitin-associated chaperon PLIC-1 (also named ubiquilin; Bedford et al., 2001; Zhang et al., 2015). Given the association of PLIC proteins with the proteasome in the ER (Kleijnen et al., 2000), it was proposed that PLIC-1 inhibits GABA_A_R degradation via ERAD (Bedford et al., 2001; Saliba et al., 2008). Accordingly, synaptic upregulation and downregulation of surface GABA_A_Rs are governed by changes in poly-ubiquitination dynamics of newly synthesized β3 subunits in the ER (Saliba et al., 2007; Fig. 2). In contrast, the ubiquitination of the γ2 subunit does not alter the forward-directed biosynthetic route, but instead triggers GABA_A_R endocytosis and subsequent lysosome-dependent degradation (Arancibia-Cárcamo et al., 2009; Jin et al., 2014; Fig. 2). In the cerebellum, the USP14-dependent deubiquitination of the α1 subunit, which is contained in the majority of cerebellar GABA_A_Rs, reduces surface GABA_A_Rs and favors receptor sorting toward lysosomal compartments (Lappe-Siefke et al., 2009; Fig. 1*C*). At inhibitory synapses, unlike their glutamatergic counterparts, no scaffolding proteins were found ubiquitinated, so far. Notwithstanding, gephyrin, the major scaffolding protein of inhibitory synapses (Tyagarajan and Fritschy, 2014), contains two PEST sequences (Tyagarajan et al., 2011). Whether these PEST motifs, as that of PSD-95, are targeted by ubiquitination is unclear.

Collectively, there is strong evidence that ubiquitination targets multiple synaptic components and represents a complex regulatory system controlling synapse formation, maintenance, and plasticity. In line with this, ubiquitination is also required for learning and memory. However, the molecular logic of how the ubiquitin system controls cognition and how targeted ubiquitination determines specific behavioral outputs remain enigmatic.

### Sumoylation

Sumoylation consists in the covalent but reversible conjugation of the 100-aa-long small ubiquitin-like modifier (SUMO) protein to specific K residues of substrates (for a more comprehensive review, see Flotho and Melchior, 2013). Like ubiquitination, sumoylation requires a dedicated enzymatic pathway and its homeostasis is finely regulated by conjugating and deconjugating enzymes. SUMO is first synthetized as a non-conjugatable precursor that is cleaved by Sentrin-proteases (SENPs) to generate a mature SUMO molecule. Mature SUMO is subsequently activated by heterodimers of SUMO-activating enzyme (SAE)1 and SAE2 in an ATP-dependent manner and transferred to the catalytic cysteine of the sole SUMO-specific conjugating enzyme UBC9. UBC9 finally catalyzes SUMO conjugation to designated substrates. Targeted K residues typically localize within the consensus motif Ψ-K-x-E, where Ψ is a hydrophobic residue and x is any aa. SUMO deconjugation occurs through the activity of SENPs. Mammalian cells express three different SUMO paralogues (*SUMO1-3*) and six SENPs (*SENP1-3*, *SENP5-7*). As all PTMs, sumoylation modulates the function of target proteins by distinct mechanisms. It can (1) trigger conformational changes that affect protein activity; (2) inhibit or favor protein-protein interactions by either generating or masking a binding site; (3) control substrate stability and turnover through a SUMO-mediated recruitment of members of the SUMO-targeted ubiquitin ligase family (Flotho and Melchior, 2013).

Originally, sumoylation was described as nuclear modification regulating protein translocation across the nuclear membrane and transcription (Matunis et al., 1996; Mahajan et al., 1997). Since then, many other SUMO substrates were identified inside and outside the nuclear compartment, including synapses. It is now well accepted that sumoylation controls several processes critical for neuronal function such as neuronal excitability and synapse development and plasticity (Schorova and Martin, 2016; Henley et al., 2018). During brain development, the SUMO machinery and neuronal sumoylome dynamics are tightly regulated in a spatiotemporal manner. In the brain, the overall expression of SUMO components and SUMO-conjugated proteins peaks during late stages of embryonic development (embryonic day (E)13–E18) and rapidly decreases postnatally (Watanabe et al., 2008; Loriol et al., 2012; Hasegawa et al., 2014; Josa-Prado et al., 2019). In addition, these proteins are redistributed from the nucleus to the synapse after birth (Loriol et al., 2012), where sumoylation still occurs in mature cortical/hippocampal neurons and is rapidly enhanced by neuronal activity (Loriol et al., 2013; Colnaghi et al., 2019). According to the critical role of sumoylation in controlling synaptic function, UBC9 and SENP1 enzymes dynamically diffuse inside and outside dendritic spines in an activity-dependent manner (Loriol et al., 2014; Schorova et al., 2019). The activation of mGlu5 triggers the transient trapping of UBC9 in the head of dendritic spines, leading to a rapid increase in synaptic sumoylation (Loriol et al., 2014). UBC9 recruitment is followed by time-dependent decrease in the exit rate of SENP1 from dendritic spines, resulting in the postsynaptic accumulation of SENP1, which restores synaptic sumoylation to initial levels (Schorova et al., 2019). Moreover, perturbation of SUMO homeostasis affects synapse physiology and consequently impacts cognitive functions. Decreasing sumoylation levels by overexpressing either a dominant negative form of UBC9 or the catalytic domain of SENP1 prevents the increase of surface AMPARs upon long-term potentiation (LTP) induction, indicating that sumoylation is essential for the expression of synaptic plasticity (Jaafari et al., 2013). Accordingly, acute inhibition of sumoylation impairs hippocampal-dependent learning and memory in mice (Lee et al., 2014). Neuron-specific silencing of *SUMO1-3* induces anxiety-like responses and impaired episodic memory providing the first evidence that SUMO conjugation is essential for emotionality and cognition (Wang et al., 2014). Conversely, increase of global SUMO1-ylation in *SUMO1* transgenic mice or by chronic infusion of exogenous SUMO1 impairs learning and memory performances (Matsuzaki et al., 2015; Yoo et al., 2017). Furthermore, the loss of *SENP2* in conditional KO mice results in several behavioral defects including spatial working memory impairment (Huang et al., 2020). RNA sequencing also revealed that the expression of genes critical for learning and memory is finely regulated by SENP2.

Together, these findings pointed out that sumoylation is essential to synapse development and function, and a balanced sumoylation/desumoylation is crucial for learning and memory processes. In line with this, over the last decade, several groups identified novel SUMO targets critical to synaptic function (Schorova and Martin, 2016; Henley et al., 2018).

#### Extrasynaptic sumoylation critical to the synaptic function

The first identified SUMO substrates bearing synaptic functions were components of the myocyte enhancer factor 2 (MEF2) family of transcription factors (Fig. 1*B*). *In vitro* and *in vivo* studies showed that sumoylation represses the transcriptional activity of MEF2A, MEF2C, and MEF2D (Grégoire et al., 2006; Kang et al., 2006; Shalizi et al., 2006). In particular, MEF2A sumoylation at K403 is critical for dendritic claw differentiation in the cerebellum via transcriptional inhibition of the transcription factor *Nur77* (Shalizi et al., 2006). Interestingly, MEF2A sumoylation is tightly modulated by neuronal activity and requires a complex cross talk with other PTMs. The Ca^2+^-dependent phosphatase calcineurin dephosphorylates MEF2A at serine (S)408 and triggers the switch of K403 from being sumoylated to acetylated, resulting in the activation of MEF2A. Acetylated MEF2A thus enhances *Nur77* transcription and leads to the inhibition of dendritic claw formation. Subsequently, it was also demonstrated that MEF2A sumoylation is critical for presynaptic differentiation by regulating the transcription of synaptotagmin 1, thus indicating that MEF2A sumoylation has a broader role in synaptic development (Shalizi et al., 2007).

A second extrasynaptic target of the SUMO machinery is the DNA binding protein and transcriptional repressor Methyl-CpG binding protein 2 (MECP2; Fig. 1*B*). Sumoylation at K223 of MECP2 is required for the recruitment of histone deacetylase complexes 1/2 (HDAC 1/2) and the consequent repression of gene expression, an essential event for the correct development of hippocampal excitatory synapses *in vitro* and *in vivo* (Cheng et al., 2014). Conversely, sumoylation at K412 decreases the physical interaction between MECP2 and the cAMP response element-binding protein (CREB), which ultimately enhances the transcription of the brain-derived neurotrophic factor (*Bdnf*) transcription (Tai et al., 2016). Similar to MEF2A, MECP2 phosphorylation in nearby residues (S421 and threonine ‘T’308) facilitates MECP2 sumoylation, further highlighting the functional interplay between distinct PTMs. As described below, mutations in *MECP2* gene are associated with neurodevelopmental disorders, among which the Rett syndrome (RTT) is the most prevalent (Guy et al., 2011).

Recently, two other transcription factors, FOXP1 and FOXP2, were found sumoylated (Fig. 1*B*). These factors are required for the assembly of cerebellar circuits underlying vocalization and motor skills (Bacon and Rappold, 2012). FOXP2 sumoylation at K674 regulates its transcriptional activity (Estruch et al., 2016; Meredith et al., 2016) and mediates dendritic outgrowth and arborization of Purkinje cells (Usui et al., 2017). In line with this, individuals from a family affected by speech and language disorders display a significant reduction of sumoylated FOXP2, supporting the pivotal role of FOXP2 sumoylation for the acquisition of these communication skills (Meredith et al., 2016). Similarly to FOXP2, FOXP1 sumoylation at K670 is indispensable for dendritic outgrowth and complexity in cortical neurons via inhibition of FOXP1 transcriptional activity (Rocca et al., 2017). Remarkably, the autism-linked *CNTNAP2* gene, which promotes the development of dendritic arbors, is transcriptionally repressed by sumoylated FOXP1, suggesting a potential molecular mechanism underlying FOXP1 function during neuronal development (Rocca et al., 2017).

Fragile X mental retardation protein (FMRP) is the most recently identified extrasynaptic SUMO target with synaptic roles (Khayachi et al., 2018; Fig. 1*B*). FMRP is an RNA-binding protein and a key component of neuronal mRNA granules. In these granules, FMRP transports translationally-repressed mRNAs along axons and dendrites to the base of active synapses, where their local translation is a key process for synapse maturation and plasticity (Prieto et al., 2020). The rapid activation of mGlu5 results in FMRP sumoylation at K88 and K130, which triggers the dissociation of FMRP from mRNA granules and enables the local translation of mRNAs that control dendritic spine elimination and maturation (Khayachi et al., 2018). It remains to be elucidated whether FMRP sumoylation discharges the whole set of mRNA molecules associated with FMRP or promotes the release of a specific subset of these mRNAs.

#### Presynaptic sumoylation

At the presynaptic compartment, sumoylation regulates diverse functions. Synaptosomal fractions loaded with purified SUMO1 display a significant reduction of Ca^2+^ influx and KCl-evoked glutamate release. Accordingly, Ca^2+^ influx and KCl-evoked glutamate release are enhanced in synapses supplemented with SENP1 (Feligioni et al., 2009). Intriguingly, modulation of synaptic sumoylation produces the reverse effect on glutamate release evoked by kainate stimulation. These results suggest that different stimuli triggers sumoylation of distinct presynaptic proteins to either inhibit or promote NT release (Feligioni et al., 2009). A primary presynaptic function controlled by the SUMO pathway is SV docking/priming through sumoylation of RIM1α and synapsin Ia (SYNIa; Fig. 1*A*). Sumoylation of SYNIa triggers its association with SVs and facilitates SV anchoring at the presynaptic membrane (Tang et al., 2015). Similarly, RIM1α sumoylation at K502 promotes its interaction with the voltage-gated Ca^2+^ channels (Ca_V_) 2.1, favoring their clustering at the presynaptic membrane and enhancing the Ca^2+^ influx required for NT release (Girach et al., 2013). Conversely, non-sumoylated RIM1α prevents Ca_V_2.1 clustering and increases SV docking in the active zone. Thus, the switch between the sumoylated and non-sumoylated forms of RIM1α is a key determinant for the fast and synchronous NT release.

Sumoylation also directly controls SV exocytosis by targeting STX1A (Craig et al., 2015; Fig. 1*A*). Sumoylated STX1A is more associated with two other components of the SNARE complex, SNAP-25 and VAMP-2, providing the mechanical forces required for SV exocytosis. Indeed, preventing STX1A sumoylation reduces its interaction with SNARE proteins and disrupts the balance of SV endo/exocytosis, skewing toward endocytosis.

In 2015, Matsuzaki and colleagues generated a transgenic mouse line overexpressing *SUMO1* in neurons aiming to identify the neuronal sumoylome (Matsuzaki et al., 2015). Among the 95 SUMO1 substrates identified by mass spectrometry (MS), only synaptotagmin-1 was further validated biochemically (Fig. 1*A*). Nevertheless, further investigations are required to exclude possible off-target sumoylation owing to SUMO1 overexpression.

Regarding mGlu receptors, the majority of Group III were identified as SUMO substrates in primary cultured neurons and heterologous cells (Tang et al., 2005; Seebahn et al., 2008; Dütting et al., 2011; Wilkinson and Henley, 2011; Fig. 1*A*). mGlu7 is the sole member shown to be sumoylated in brain tissues. Like other SUMO targets, its sumoylation has to be preceded by PKC-dependent phosphorylation at S862 (Choi et al., 2016). Opposed to ubiquitination, mGlu7 sumoylation stabilizes its expression at the cell surface. Therefore, ubiquitination and sumoylation of mGlu7 bidirectionally modulate its surface expression at the presynaptic membrane and ultimately, regulate NT release. The type-1 endocannabinoid (CB1) receptors are another class of metabotropic receptors that regulates presynaptic functions (Castillo et al., 2012). Interestingly, it was shown that conjugated and unconjugated SUMO1 levels transiently increase upon CB1 activation as well as CB1 itself might be targeted by SUMO (Gowran et al., 2009). However, additional experiments are needed to confirm this hypothesis.

Among the voltage-gated ion channels targeted by sumoylation, potassium channels are the most represented (Fig. 1*A*). *Senp2*-deficient mice display hypersumoylation of voltage-gated potassium (K_V_) 1.1 and 7.2/7.3 channels. While sumoylation of K_V_1.1 does not alter channel activity, enhanced sumoylation of K_V_7.2 reduces depolarizing M-currents, resulting in neuronal hyperexcitability and epileptic seizures (Qi et al., 2014). Similarly, sumoylation of surface K_V_2.1 reduces channel activity and, as a consequence, enhances neuronal excitability (Plant et al., 2011). Interestingly, K_V_4.2 was recently found sumoylated in two distinct sites, producing different effects (Welch et al., 2019). While sumoylation at K437 triggers K_V_4.2 surface expression, sumoylation at K579 decreases the amplitude of K currents without any change in K_V_4.2 trafficking. Differently, the sumoylation of voltage-gated sodium (Na_V_) 1.2 channels enhances the amplitude of Na^+^ currents (Plant et al., 2016; Fig. 1*A*).

Sumoylation is also essential to control the axonal trafficking and function of Na_V_1.7 by targeting the collapsin response mediator protein 2 (CRMP2; Fig. 1*A*). CRMP2 is highly expressed in the brain and is critical for microtubule remodeling, neuronal polarity, axon outgrowth, and synapse dynamics (Arimura et al., 2004; Zhang et al., 2016). Expression of a CRMP2 SUMO-deficient mutant prevents its physical interaction with Na_V_1.7 and decreases Na_V_1.7 surface expression, resulting in reduced amplification of membrane depolarization in presynaptic boutons (Dustrude et al., 2013, 2017; Ju et al., 2013). CRMP2 sumoylation is also phosphorylation dependent (Dustrude et al., 2016). Indeed, interfering with either CRMP2 sumoylation or phosphorylation reduces surface Na_V_1.7. CRMP2 also interacts with the actin cytoskeleton in dendrites and dendritic spines. It was recently found that desumoylation and dephosphorylation of CRMP2 independently promote the formation and maturation of dendritic spines, broadening the importance of PTMs in regulating the multiple functions of CRMP2 (Zhang et al., 2018).

Beside targeted sumoylation, an intriguing concept that might also apply to synapses (Henley et al., 2018) is that sumoylation may occur simultaneously to components of protein assemblies. This idea of a synchronous “SUMO spray” on multiple targets (named “SUMO velcro”) was first shown to occur in the nucleus, where DNA damage triggers a SUMO wave that activates multiple components of the enzymatic pathway required to repair the DNA (Psakhye and Jentsch, 2012). Although not yet experimentally validated, the model of SUMO spray might properly suit to presynaptic NT release, which requires a coordinated series of protein-protein interactions that are tightly regulated by sumoylation (Henley et al., 2018).

#### Postsynaptic sumoylation

To date, postsynaptic sumoylation has been less characterized than extrasynaptic and presynaptic sumoylation. Nevertheless, the first identified synaptic target of the SUMO pathway was GluK2 (Martin et al., 2007; Figs. 1*A*, 2). Kainate stimulation promotes GluK2 sumoylation at K886, triggering receptor endocytosis during LTD expression at mossy fiber–CA3 synapses in the hippocampus (Martin et al., 2007; Konopacki et al., 2011; Chamberlain et al., 2012). As for other SUMO targets, the PKC-dependent phosphorylation of S868-GluK2 is required for its subsequent sumoylation (Konopacki et al., 2011; Chamberlain et al., 2012). As aforementioned, GluK2 is also ubiquitinated. However, whether these two PTMs operate in synergy, reciprocally compete or are independent remains to be investigated.

A second postsynaptic SUMO target is the calcium/calmodulin-dependent serine protein kinase (CASK; Fig. 1*A*). CASK belongs to the membrane-associated guanylate kinase (MAGUK) family of scaffolding proteins and is involved in dendritic spine formation and maturation through the modulation of the actin cytoskeleton via the interaction with the protein 4.1 (Biederer and Südhof, 2001). The protein 4.1 binds spectrin and bridges the association between F-actin and spectrin, stabilizing actin in dendritic spines. Conjugation of SUMO1 to K679 of CASK interferes with the interaction between CASK and protein 4.1 and, consequently, regulates spine density and morphology in developing neurons (Chao et al., 2008).

Among postsynaptic signaling proteins, ARC, which is also ubiquitinated, is the sole SUMO target identified so far (Craig and Henley, 2012; Craig et al., 2012; Figs. 1*A*, 2). The expression of a constitutively desumoylated ARC selectively prevents the increase of surface AMPARs during TTX-induced synaptic up-scaling, indicating that sumoylation inhibits ARC function. Interestingly, the same treatment also augments the amount of SUMO1-conjugated proteins and decreases SENP1 levels (Craig et al., 2012). Recently, it was demonstrated that sumoylated ARC is more broadly implicated in synaptic plasticity. During LTP consolidation, sumoylated ARC selectively accumulates in synaptosomal and cytoskeletal fractions, where it forms a complex with the F-actin binding protein drebrin A, stabilizing nascent actin filaments in dendritic spines (Nair et al., 2017). These results further support the role of sumoylation as master regulator of different forms of synaptic plasticity.

At inhibitory synapses, gephyrin is extensively subjected to PTMs (Tyagarajan and Fritschy, 2014). In 2016, it was shown that gephyrin sumoylation at K148 and K724 negatively regulates its postsynaptic clustering and, ultimately, reduces synapse formation and inhibitory transmission (Fig. 1*C*). Gephyrin sumoylation is intimately linked to other PTMs, which synergistically operate to orchestrate its clustering dynamics (Ghosh et al., 2016). Desumoylation of gephyrin at K148 leads to deacetylation at K666 and dephosphorylation at S268 residues, enhancing its postsynaptic clustering and the assembly of inhibitory synapses.

Recently, the presence of SUMO1-conjugated proteins at synapses was questioned, bringing out an intense scientific debate on this topic (Tirard et al., 2012; Daniel et al., 2017, 2018; Wilkinson et al., 2017). By using KI mice expressing His_6_-HA-SUMO1, Dr. Brose and Dr. Tirard teams were not able to validate sumoylation of seven previously characterized presynaptic and postsynaptic SUMO targets nor to observe SUMO1 conjugation at synapses (Daniel et al., 2017). On the contrary, they could confirm that extra-synaptic/nuclear sumoylation is present (Tirard et al., 2012), concluding that there is no evidence of synaptic SUMO1-ylation. Notwithstanding, several arguments support the presence of SUMO1-ylation at the synapse questioning whether this animal model is suitable to study sumoylation. (1) In His_6_-HA-SUMO1 KI mice, there is 20–30% less SUMO1 conjugation than WT mice, and this impairment is associated to a significant increase of SUMO2/3-conjugated proteins (Tirard et al., 2012; Daniel et al., 2017). This reduction in the efficiency of SUMO1 conjugation could make particularly challenging the detection of SUMO targets in the synaptic compartment where sumoylation is already low and extremely transient; (2) a wealth of studies confirmed the functional relevance of synaptic sumoylation (e.g., use of non-sumoylatable mutants), while Daniel and colleagues did not carry out this analysis (for review, see Henley et al., 2018; Schorova and Martin, 2016); (3) consistent data clearly indicate the presence of components of the SUMO machinery at synaptic sites (Watanabe et al., 2008; Loriol et al., 2012, 2014; Hasegawa et al., 2014; Josa-Prado et al., 2019; Schorova et al., 2019); (4) some key technical differences may explain the failure to detect synaptic sumoylation (e.g., weak SUMO1 immunostaining and the use of antibodies that do not recognize sumoylated proteins). Although in our opinion substantial data indicate the presence and the functional relevance of synaptic SUMO1-ylation, the aforementioned studies pointed out the need of defining strict consensus criteria to investigate sumoylation in the brain.

### Neddylation

Neddylation consists in the covalent and reversible attachment of a NEDD8 (for neural precursor cell expressed, developmentally downregulated 8) moiety to a K residue of target proteins. Of the UBLs, NEDD8 shows the greatest degree of similarity with ubiquitin (∼80%). Like ubiquitination and other UBLs, neddylation requires a three-step enzymatic cascade to activate and covalently attach NEDD8 to K residues of target proteins (Enchev et al., 2015). While E1 activating and E2 conjugating enzymes have been identified (NEDD8 activating enzyme, NAE as E1, and UBC12 and UBE2F as E2 enzymes), the E3 ligases are as yet ill-defined. Deconjugation of NEDD8 from targets is achieved by NEDD8-specific proteases, namely the metalloprotease CSN5, the cysteine protease NEDP1 and USP21 (Enchev et al., 2015).

Although NEDD8 function and regulation remain largely unexplored, recent papers shed light on the importance of neddylation in synaptic maturation, function and plasticity. Neddylation is observed during embryonic brain development, its expression increases during the first two postnatal weeks and is then maintained throughout adulthood (Kumar et al., 1992; Vogl et al., 2015). As the best characterized function of NEDD8 involves the activation of ubiquitin E3 ligases of the Cullin-RING family (Fig. 1*A*), neddylation is thought a major regulator of ubiquitination (Enchev et al., 2015). In the hippocampus, NMDAR-dependent neddylation promotes the UPS-dependent degradation of the DNA methyltransferase DNMT3a1 leading to demethylation of the BDNF promoter and enabling BDNF expression during memory consolidation (Fig. 1*A*; Bayraktar et al., 2019). Among the members of the cullin-RING family, several ligases mediate the targeted degradation of synaptic elements and negatively regulate synapses (e.g., cullin3 and parkin). However, inhibition of the neddylation pathway does not enhance synaptic activity and strength as it would have been expected as a consequence of reduced synaptic ubiquitination. Instead, it destabilizes dendritic spines, affects synaptic transmission and plasticity, and impairs cognitive functions (Scudder and Patrick, 2015; Vogl et al., 2015; Brockmann et al., 2019), thus suggesting that neuronal neddylation targets other substrates relevant to synaptic function. In line with this, Vogl and colleagues elegantly demonstrated that PSD-95 is neddylated at K202 by MDM2 (Vogl et al., 2015), which also mediates PSD-95 ubiquitination (Fig. 1*A*; Colledge et al., 2003; Bianchetta et al., 2011). Although PSD-95 neddylation does not modify its ubiquitination and degradation rate, neddylation reduces synaptic clustering of PSD-95. Furthermore, the expression of a non-neddylatable mutant recapitulates at least some of the molecular and cellular phenotypes observed with PSD-95 knock-down (Ehrlich et al., 2007), indicating that NEDD8-conjugation is required for PSD-95 proactive functions. PSD-95 is a major molecular hub of excitatory synapses and mediates multiple interactions with several proteins (Sheng and Kim, 2011). How neddylation changes PSD-95 conformation and/or affinities with its partners remains to be uncovered. Very recently, a NEDD8-ubiquitin substrate profiling detected 341 neddylated proteins in HEK293 cells expanding the repertoire of neddylated targets and indicating broader roles of neddylation than the sole activation of cullin-RING E3 ligases (Vogl et al., 2020). Among the identified targets, the authors biochemically and functionally characterized cofilin neddylation in the brain and found that it is required to ensure proper dendrite development in mouse cortical neurons (Fig. 1*A*; Vogl et al., 2020).

## ID and Impairment of Ubiquitination and Sumoylation Pathways

ID is a neurodevelopmental disorder with an estimated prevalence of 1–3%. The formal diagnosis of ID is based on the intelligence quotient (IQ) test, which should be scored <70, and the presence of deficits in at least two adaptive behaviors that affect everyday activities. It is classified as mild, moderate, severe, or profound based on IQ score. ID is defined as non-syndromic if the intellectual deficit is the sole clinical feature, or as syndromic if the mental impairment is comorbid with other neurologic pathologies such as epilepsy, sensory alterations and ASD (Ilyas et al., 2020). ID is a complex multifactorial disease, in which environmental and genetic factors and their reciprocal interaction critically contribute to its etiology. Yet, 25% of ID cases have clear genetic origins and some of these forms are monogenic. The environmental factors that underlie ID comprise stressful events occurring at early stages of neurodevelopment, including drug and alcohol abuse and infections during pregnancy, birth complication, and severe malnutrition. Thanks to recent advances in whole-genome sequencing, the identification of genetic defects is becoming increasingly efficient. To date, ∼700 genes were associated to either syndromic or non-syndromic ID. Notably, more than 50% of these genes encode presynaptic or postsynaptic proteins, or proteins implicated in synapse development, function and plasticity (Ilyas et al., 2020). Among the several genes associated with ID, ∼100 genes are located on chromosome (chr.) X and are responsible for X-linked ID (XLID; Ropers and Hamel, 2005).

Numerous ID-associated genes code for proteins targeted by ubiquitination and sumoylation or components of ubiquitin and SUMO machineries. Furthermore, some extrinsic risk factors of ID seem to be associated with alterations of brain ubiquitome and/or sumoylome. As described above, several studies indicate that ubiquitination and sumoylation are critical to the expression of synaptic plasticity, the cellular correlate of learning and memory processes and whose impairment is a major hallmark of ID (Aicardi, 1998). Collectively, this evidence suggests that ubiquitination and sumoylation failure may be implicated in ID pathogenesis. Despite pharmacological or genetic blockade of neddylation results in cognitive dysfunctions that may manifest in ID patients (e.g., impaired memory and sociability; Vogl et al., 2015), current evidence does not indicate a direct involvement of neddylation in the development of ID. Below, we provide an overview of the ID forms that are linked to defective ubiquitin and SUMO pathways.

### Ubiquitination and ID

Beyond the well-established role of ubiquitination in synapse physiology, its dysregulation has been largely linked to synaptic dysfunction and ID pathogenesis (Mabb and Ehlers, 2010; Upadhyay et al., 2017; George et al., 2018). As listed in Table 1, causal genes directly encode either components of the ubiquitin machinery or regulators of ubiquitination (*PLAA* and *MAGEL2* genes). A third category, not discussed here for space constraints comprises genes encoding targets of ubiquitination in which pathogenic mutations may change their ubiquitination profile. The molecular mechanisms underlying the function of the first two categories of genes and the neuronal substrates that they target are poorly characterized. For these reasons, the molecular pathogenesis of these forms of ID is unclear.

**Table 1:** Rare monogenic forms of ID linked to mutations of components or regulators of the ubiquitin pathway

Gene	Protein	Disease	Genetic abnormalities	References
*ASXL3*	Component of the Polycomb repressive deubiquitination (PR-DUB) complex	Bainbridge–Ropers syndrome, BRS (OMIM 615485) and ASD	*De novo* truncating mutations in BRS and missense mutations in ASD	Bainbridge et al. (2013); De Rubeis et al. (2014)
*CUL4B*	Scaffolding protein stabilizing cullin RING E3 ligase	XLID (OMIM 300304)	Missense mutations	Wang et al. (2013); Zou et al. (2007)
*HECW2*	E3 ubiquitin ligase HECW2	Neurodevelopmental disorder with hypotonia, seizures, and absent language, NDHSAL (OMIM 617268)	Missense mutations	Halvardson et al. (2016)
*HERC1*	E3 ubiquitin ligase HERC1	Macrocephaly, dysmorphic facies, and psychomotor retardation, MDFPMR (OMIM 617011)	Missense and frameshift mutations	Aggarwal et al. (2016); Nguyen et al. (2016); Ortega-Recalde et al. (2015)
*HERC2*	E3 ubiquitin ligase HERC2	Syndrome of ID, autism, and variable neurological deficits (OMIM 615516)	Missense mutations	Puffenberger et al. (2012)
*HUWE1*	HECT, UBA, and WWE domain containing 1, E3 ubiquitin protein ligase	Turner type, XLID (OMIM 309590)	Microduplications, missense mutations	Froyen et al. (2008); Moortgat et al. (2018)
*MAGEL2*	E3 ubiquitin ligase enhancer	Prader–Willi syndrome, PWS (OMIM 176270) and Schaaf–Yang syndrome, SHFYNG (OMIM 615547)	Interstitial deletions and maternal uniparental disomy in PWS; truncating mutations in SHFYNG	Tacer and Potts (2017)
*MID2*	Member of the TRIpartite motif (TRIM) family of RING E3 ligases	XLID (OMIM 300928)	Missense mutation	Geetha et al. (2014)
*OTUD6B*	Member of the ovarian tumor domain (OTU)-containing subfamily of deubiquitinating enzymes	Intellectual developmental disorder with dysmorphic facies, seizures, and distal limb anomalies, IDDFSDA (OMIM 617452)	Truncating and missense mutations	Santiago-Sim et al. (2017)
*PLAA*	Ubiquitin binding protein phospholipase A2 activating protein	Neurodevelopmental disorder with progressive microcephaly, spasticity, and brain anomalies, NDMSBA (OMIM 617527)	Missense mutations	Falik Zaccai et al. (2017); Hall et al. (2017)
*RLIM*	RNF12 E3 ubiquitin ligase	Tonne–Kalscheuer syndrome, XLID (OMIM 300978)	Missense mutation	Tønne et al. (2015)
*TRIM50*	Member of the TRIpartite motif (TRIM) family of RING E3 ligases	William–Beuren syndrome, WBS (OMIM 194050)	Microdeletion on chr. 7q11.23	Micale et al. (2008)
*TRIP12*	Member of the HECT domain E3 ubiquitin ligases family	ID with or without ASD (OMIM 6177520)	CNVs, missense, frameshift, splicing mutations	Zhang et al. (2017)
*UBE2A*	E2 ubiquitin conjugating enzyme E2A	XLID type Nascimento (OMIM 300860)	Truncating and missense mutations	Budny et al. (2010); Nascimento et al. (2006)
*UBE3B*	E3 ubiquitin ligase E3B	Blepharophimosis-ptosis-ID syndrome, BPIDS (OMIM 244450)	Truncating mutations	Basel-Vanagaite et al. (2012)
*USPX9*	Deubiquitinating enzyme FAF-X	Female-restricted X-linked non-syndromic mental retardation-99 (OMIM 300919)	Truncating mutation and X-chr. deletion	Au et al. (2017)

XLID: X-linked ID, CNV: copy number variation.

In general, ubiquitin-linked ID forms present comorbidities that are commonly present in ID. Mental impairment is often accompanied by dysmorphic features and multiple neurologic and neuropsychiatric manifestations (e.g., epilepsy, motor dysfunction, and autistic behaviors). Many syndromes are also multisystem disorders underscoring the ubiquitous importance of ubiquitination for tissue and body formation and function. To the best of our knowledge, a clear classification of ID forms, and more generally of brain disorders linked to defective ubiquitination, does not exist. Functional and genetic groups may be however elaborated. For instance, a genetic classification may be based on components of the enzymatic cascade mediating the different steps of ubiquitin conjugation/deconjugation (e.g., E2-E3, and DUB enzymes). Functional classes may include ubiquitinating enzymes involved in distinct cellular functions, such as regulation of transcription, trafficking, cell division or proteasome activity. Although other criteria may certainly be applied to propose alternative classifications including comorbidities, no unique behavioral phenotypes associate with any of the aforementioned groups. Overall, it clearly emerges that ubiquitination and its delicate homeostasis are fundamental for brain physiology. Indeed, the perturbation of this equilibrium caused by the altered expression or activity of a single E3 ubiquitin ligase is hardly compensated by the other ∼600 E3 and is sufficient to lead to severe pathologic states of the brain. Here, we discuss in more details the implications of defective ubiquitination in the molecular pathogenesis of two of the most common forms of syndromic ID, the AS and Down syndrome (DS).

#### Angelman syndrome

AS (OMIM 105830) is a relatively rare neurodevelopmental disorder (prevalence of 1/15,000 live births) characterized by severe intellectual deficit, motor dysfunction, unusually happy demeanor, seizures and autism-like behavior (Clayton-Smith and Laan, 2003; Margolis et al., 2015; Buiting et al., 2016). AS results from distinct genetic abnormalities that ultimately lead to the loss of the brain-specific imprinted *UBE3A* (ubiquitin protein ligase E3A, also referred to as E6AP) gene (Mabb et al., 2011). *UBE3A* encodes three isoforms that are generated by alternative splicing and localize either in the nucleus or cytosol (Miao et al., 2013). In the cytosol, UBE3A is found in axonal terminals and dendritic spines (Dindot et al., 2008; Burette et al., 2018). While *UBE3A* loss results in AS, elevated expression or activity of *UBE3A* represent the most identifiable genetic form of ASD, indicating that a tight balance in the dosage of this gene is critical to develop functional neuronal circuits.

Data from AS mouse models suggest that UBE3A plays a key role in modulating synaptic pathways important for cognition and behavior. Neurons from *Ube3a*-KO mice display abnormal dendritic spines, reduced excitatory neurotransmission and impaired synaptic plasticity (Dindot et al., 2008; Yashiro et al., 2009; Pignatelli et al., 2014; Kim et al., 2016; Avagliano Trezza et al., 2019; Wang et al., 2019a). Given the primary function of UBE3A as E3 ubiquitin ligase, defective ubiquitination is thought to be at the basis of neuronal dysfunctions and clinical manifestations of AS. However, only a few substrates have been identified and shown to be functionally relevant to AS etiology. For these reasons, the molecular underpinnings of UBE3A function remain enigmatic. Different mechanisms, mainly driven by the defective ubiquitination hypothesis, have been put forward on the pathogenic role of UBE3A. (1) In physiological conditions, UBE3A may indirectly modulate the number of AMPARs at the postsynaptic membrane by ubiquitinating the aforementioned signaling protein ARC and promoting its degradation (Greer et al., 2010). Therefore, loss of UBE3A may increase ARC levels and lead to enhanced AMPAR internalization and, ultimately, depression of glutamatergic transmission. (2) A second synaptic substrate of UBE3A is ephexin 5 (Margolis et al., 2010). Ephexin 5 is a guanine nucleotide exchange factor (GEF) activator of the RhoA GTPase that limits the number of excitatory synapses via inhibition of the synaptogenic, *trans*-synaptic EphB receptor/ephrin ligand complex (Henderson and Dalva, 2018). *Ube3a*-KO mice display increased amounts of ephexin 5 and abnormal density of excitatory synapses, indicating that UBE3A-dependent degradation of ephexin 5 is critical for the normal development of excitatory synapses. (3) A recent study suggested that UBE3A ubiquitinates the small conductance potassium channel SK2, whose major function is to repolarize neuronal membranes after depolarization (Ngo-Anh et al., 2005) and facilitates its endocytosis (Sun et al., 2015b). In line with defective ubiquitination of SK2, its surface expression is significantly increased in *Ube3a*-KO mice, resulting in membrane hyperpolarization and impaired synaptic plasticity. (4) A role for CAMKII was proposed based on the observation that hippocampi derived from AS mice display reduced CAMKII activity and excessive inhibitory autophosphorylation (Weeber et al., 2003). In particular, the correction of some AS neurologic deficits obtained preventing CAMKII inhibitory autophosphorylation suggests that CAMKII may be instrumental to UBE3A-dependent pathways underlying AS phenotype (van Woerden et al., 2007). However, it is not known whether UBE3A directly targets CAMKII for degradation or targets unknown kinases or phosphatases that in turn modulate CAMKII phosphorylation. Strikingly, PTPA, which is the activator of a major phosphatase of CAMKII, PP2A, was recently identified as UBE3A target (Wang et al., 2019a). However, the modulation of PTPA levels does not affect the phosphorylation of CAMKII, suggesting other downstream substrates. (5) In cerebellar Purkinje cells of AS mice, mechanistic target of rapamycin (mTOR) signaling is unbalanced, resulting in upregulated mTORC1 and reduced mTORC2 (Sun et al., 2015a). mTORC1 activation relies on its lysosomal recruitment through the interaction with the Rag GTPase-Ragulator complex. UBE3A ubiquitinates the p18 subunit of the Ragulator complex. Therefore, in AS mouse models defective p18 ubiquitination enhances mTORC1 activity by increasing its lysosomal recruitment. (6) A recent proteomic-based study aimed at identifying new UBE3A substrates implicated autophagy in AS pathogenesis (Wang et al., 2019b). UBE3A physically associates with and ubiquitinates the autophagy regulator Huntington-associated protein 1 (HAP1), a key protein for autophagosome trafficking. In AS mouse model, increased HAP1 leads to excessive autophagy and ultimately, dendritic spine defects. Consistently, pharmacological attenuation of autophagy partially alleviates synaptic dysfunction and behavioral deficits in AS mice.

The above studies have focused on mechanisms underlying weakened excitatory synapses. However, the vast majority of AS patients display autistic behavior and seizure susceptibility (Clayton-Smith and Laan, 2003), suggesting that loss of UBE3A may increase the excitation (E)/inhibition (I) ratio in the neocortex. Recent evidence proposed that UBE3A loss might impact GABAergic neurons more severely than the excitatory counterpart, with the potential net outcome favoring hyperexcitability (Wallace et al., 2012; Judson et al., 2016). Future studies aimed at investigating whether UBE3A directly operates at inhibitory synapses and which are its substrates will help clarifying UBE3A-mediated mechanisms modulating neuronal excitability.

Together, these studies clearly indicate that UBE3A regulates several cellular pathways that critically contribute to synapse development and function, and that altered ubiquitination is central to AS etiology. Although the number of identified UBE3A substrates is rapidly increasing, the functional relevance of these interactions is not clear yet and an integrated picture of UBE3A function is lacking. In addition, the weight of individual substrates and pathways in the pathogenesis of AS and its clinical manifestations is still enigmatic.

#### Down syndrome

DS (OMIM 190685) is the most common cause of ID and accounts for ∼15–20% of all individuals affected by ID. DS is a complex genetic disorder characterized by heterogenous clinical manifestations. Among them, cognitive impairment is present in all DS individuals (Antonarakis et al., 2004). The DS critical region (DSCR) is a relatively small locus on chr.21, and its triplication is necessary and sufficient to generate DS cognitive deficits. One of the genes localized in the DSCR is *TTC3*, which is consistently upregulated in patients and animal models of DS (Saran et al., 2003). *TTC3* encodes an E3 ubiquitin ligase that targets a phosphorylated form of the kinase AKT (Suizu et al., 2009), which is critical for several cellular functions in the brain (Hers et al., 2011). In *Ts65Dn* mice, the most common animal model of DS, the abnormal levels of TTC3 enhance ubiquitination and degradation of AKT. Among its multiple synaptic functions, AKT phosphorylates the β subunits of GABA_A_Rs in hippocampal neurons, thereby increasing the number of surface GABA_A_Rs at inhibitory synapses (Wang et al., 2003). Remarkably, DS individuals show a higher incidence of seizures, raising the possibility that enhanced TTC3-mediated degradation of AKT leads to the loss of E/I equilibrium in the brain and hyperexcitability. In developing neurons, *TTC3* overexpression also limits neurite outgrowth and modifies the morphology of the Golgi apparatus through the modulation of actin-regulating pathways (Berto et al., 2014). Strikingly, *TTC3* also significantly correlates with other DS-unrelated brain pathologies associated with ID, suggesting an essential role for *TTC3* in complex cognitive functions (Vilardell et al., 2011).

Dysfunctions of global neuronal ubiquitome in DS are also detected in aged *Ts65Dn* mice and postmortem brains of DS patients. Such alterations are linked to the accumulation of inclusion bodies in the cerebellum, a cellular hallmark of Alzheimer’s disease (AD), a neurodegenerative disorder that is particularly frequent in DS (Necchi et al., 2011; Tramutola et al., 2017). On the other hand, it was reported that in both DS patients and *Ts65Dn* mice the *USP16* gene, which maps on chr.21 and codes for a deubiquitinase, is critical to DS pathogenesis (Adorno et al., 2013). *USP16* triplication excessively removes ubiquitin from histone H2A, a key event for the self-renewal and expansion of different progenitor cells, including neuronal progenitors. In line with this, a reduced expansion of postnatal neuronal progenitors is observed in DS patients.

All of these pieces of evidence suggest that unbalanced ubiquitination critically contributes to neuronal circuit dysregulation and clinical manifestations of DS, including ID. The relative contribution of these pathways to the molecular pathogenesis of DS remains largely unknown.

### Sumoylation and ID

As aforementioned, sumoylation is critical to build up proper synaptic connections by modulating the function of several neuronal proteins. The evidence that several SUMO targets are ID-associated proteins raises the possibility that impaired sumoylation may be relevant to ID etiology. An increasing number of molecular studies now supports this hypothesis and suggests that dysregulated neuronal sumoylation may participate to the development of two common syndromic forms of ID, the RTT and DS. Furthermore, defective sumoylation may potentially be implicated in other ID forms, such as Fragile X syndrome (FXS), Phelan–McDermid syndrome (PMS) and the Cc2d1-dependent non-syndromic ID.

#### Rett syndrome

RTT (OMIM 312750) is a devastating neurodevelopmental disorder and the leading cause of genetic ID in young girls. Almost 95% of RTT individuals carry mutations in the *MECP2* gene. As described above, MECP2 is sumoylated at K223 and K412 residues and these modifications regulate the activity of MECP2 as transcriptional repressor. The analysis of sumoylation profiles of seven different mutated *MECP2* variants identified in RTT patients reveals that six of these mutations decrease MECP2 sumoylation because of a lower affinity for the SUMO E3 ligase PIAS1 (Tai et al., 2016). Strikingly, while the lentiviral expression of either WT or sumoylated MECP2 in conditional *Mecp2*-KO mice restores some cellular and behavioral deficits, such as social interaction, fear memory and LTP, the expression of a non-sumoylatable MECP2 mutant fails to rescue these phenotypes. In this study, it was also demonstrated that these pathologic mutations are associated with altered MECP2 phosphorylation at S421, a PTM that is known to promote its sumoylation. In the same study, the authors also demonstrated that mice expressing non-sumoylatable MECP2 display 12-fold decrease in *Wnt6* mRNA levels compared to WT animals. Very recently, the same group showed that lentiviral expression of WNT6 rescues defective MECP2 sumoylation in MECP2 T158A mouse model of RTT. *Wnt6* transduction and enhanced MECP2 sumoylation also resulted in partial amelioration of locomotor and social behavioral deficits (Hsu et al., 2020). Together, these pioneer studies provided for the first time a link between altered sumoylation and the etiology of ID, representing an important milestone in the field.

#### ID linked to SUMO machinery-encoding genes

Since the *SUMO3* paralogue gene is located in the long arm of chr.21, the excessive dosage of *SUMO3* is an intriguing hypothesis in DS pathogenesis. Several transcriptional factors and the glucocorticoid receptor, which are known to control cognitive functions, are targeted by SUMO3 (Schorova and Martin, 2016). In line with this, the levels of free and conjugated SUMO2/3 proteins are significantly higher in hippocampal lysates derived from DS patients compared with healthy individuals (Gardiner, 2006). Yet, a comprehensive view of SUMO3 neuronal targets is lacking and the impact of *SUMO3* triplication on DS etiology remains elusive. Recently, Binda and colleagues reported a marked decrease of the SUMO2/3-specific deconjugating enzyme SENP3 in DS patients without any corresponding increase of SUMO2/3-conjugated proteins (Binda et al., 2017). Future studies aiming at systematically analyzing SUMO3 substrates in the brain will be of invaluable help to elucidate the role of enhanced sumoylation in DS pathogenesis.

A woman with profound developmental delay associated with several other clinical manifestations was found to carry a microdeletion in the chr.19 that resulted in the haploinsufficiency of 15 genes, including *SAE1*, a subunit of the SUMO1 activating enzyme (Leal et al., 2009). Although the authors suggested that the loss of *SAE1* gene may correlate with an impaired development of the cleft lip and palate, it remains to be determined whether this alteration might also participate to other pathologic features, comprising defective cognitive functions.

*ZMIZ1* gene codes for an androgen receptor coactivator that functions as an E3 SUMO ligase (Sharma et al., 2003) and recently identified mutations were associated with ID and developmental delay (Carapito et al., 2019). Notably, all identified *ZMIZ1* mutations occur in the SUMO acceptor site, suggesting that they might compromise the E3 SUMO ligase activity of ZMIZ1, resulting in a failure of sumoylation homeostasis in neurons.

#### Cc2d1-dependent non-syndromic ID

Mutations in the Coiled-coil and C2 domain containing 1A (*CC2D1A*) gene are associated with non-syndromic ID (OMIM 608443). This gene codes for CC2D1A, a DNA binding protein that regulates multiple cellular signaling pathways, including serotonin and dopamine receptors, dendritic arborization, synapse maturation, and plasticity (Zhao et al., 2011; Al-Tawashi et al., 2012; Manzini et al., 2014; Oaks et al., 2017; Yang et al., 2019). Consistent with these roles, the loss of CC2D1A *in vivo* determines the appearance of cognitive and social deficits (Oaks et al., 2017). Very recently, it was shown that conditional deletion of *Cc2d1a* in excitatory neurons of the forebrain leads to decreased levels of SENP1 and SENP3. As a consequence, the desumoylation of the small GTPase RAC1, one of the major targets of SENP1 and SENP3, is suppressed (Yang et al., 2019). Since RAC1 activity is enhanced by sumoylation, hyperactived RAC1 results in abnormal dendritic morphology and synaptic dysfunction. Remarkably, pharmacological blockade of RAC1 activity partially rescues deficits in synaptic plasticity and memory observed in *Cc2d1a*-cKO mice (Yang et al., 2019). In conclusion, this work provides further experimental evidence supporting the hypothesis that aberrant sumoylation is a key molecular determinant underlying ID etiology.

#### mGlu5-related IDs: Phelan–McDermid and Fragile X syndromes

PMS and FXS are two major forms of ID and are associated with dysregulation of the mGlu5-dependent pathway. The loss of *SHANK3* gene, which is caused by deletions of the terminal end of chr.22 long arm, is critical to PMS development. SHANK3 is a major scaffolding protein of excitatory synapses that anchors, together with HOMER1, mGlu5 to the postsynaptic membrane. As a consequence, the loss of Shank3 impairs mGlu5 activity by destabilizing the pool of surface receptors (Vicidomini et al., 2017). Conversely, FXS mouse models carrying loss-of-function mutations of *Fmr1* gene are characterized by excessive mGlu5-dependent signaling (Prieto et al., 2020).

Considering the functional relevance of mGlu5 to SUMO homeostasis (Loriol et al., 2014; Schorova et al., 2019), the analysis of brain sumoylome in animal models of PMS and FXS is of primary importance and might uncover pivotal roles of sumoylation in the molecular pathogenesis of these neurodevelopmental disorders. Recently, new evidence further supported the possibility that sumoylation might be a key determinant in the pathogenesis of FXS (Khayachi et al., 2018; Prieto et al., 2020). The novel pathologic missense mutation R138Q of *FMR1* gene was identified in three unrelated individuals presenting developmental delays, ID, and seizures (Collins et al., 2010; Diaz et al., 2018; Sitzmann et al., 2018). In contrast to other FXS cases, this mutation does not affect the expression levels of FMRP protein. As the R138Q mutation localizes in close proximity to the SUMO site K130 of FMRP, it is possible that the R138Q mutation causes aberrant FMRP sumoylation, and this impairment may be causative of FXS. Similarly, the F126S mutation, which was reported in a FXS male patient presenting ID and autistic features (Quartier et al., 2017), also localizes nearby K130 residue. As this mutation replaces a phenylalanine (F) with an S residue, it likely generates a novel phosphorylation site, which could subsequently promote sumoylation at K130 (Loriol et al., 2014). Further studies are required to better explore this hypothesis.

### Functional Interplay Between Ubiquitination and UBLs

Recently, the existence of a dynamic interplay between ubiquitination and UBLs provided an intriguing perspective that could lead to the identification of novel principles underlying synapse development and function. As mentioned above, the best characterized function of NEDD8 is to enhance the activity of Cullin E3 ligases providing evidence of a direct interaction with ubiquitination (Enchev et al., 2015). Moreover, neddylation-dependent ubiquitination and degradation of DNMT3a1 is critical to increase BDNF expression and is required to memory consolidation in the hippocampus (Bayraktar et al., 2019). PSD-95 is targeted by the E3 ligase MDM2, which mediates both ubiquitination and neddylation (Colledge et al., 2003; Bianchetta et al., 2011; Vogl et al., 2015). However, neddylation does not change PSD-95 degradation rate (Vogl et al., 2015), and the possible cross talk between these two PTMs on PSD-95 remains unknown. Altogether, more study is needed to better understand neddylation and its interplay with other PTMs.

Emerging evidence also indicates that ubiquitination and sumoylation reciprocally interact to precisely regulate protein function. Numerous neuronal substrates are targeted by both ubiquitin and SUMO machineries. Proteomic approaches showed that ∼25% of SUMO-acceptor K residues are also ubiquitinated, suggesting that these two pathways likely compete with each other for the same K residues (Tammsalu et al., 2014). However, a few examples suggest that this interplay is not only antagonistic, but ubiquitination and sumoylation can also operate in synergy. The hypoxia-inducible factor 1 α (HIF1α) requires to be sumoylated for its subsequent UPS-dependent degradation (Cheng et al., 2007). Similarly, the sequential sumoylation and ubiquitination of the serine hydroxymethyltransferase 1 (SHMT1) and the regulatory subunit of the kinase IKK are required to control the rate of nuclear import/export of these proteins and, ultimately, to regulate their activity (Huang et al., 2003; Anderson et al., 2012). Moreover, the impairment of ubiquitination/sumoylation cross talk critically contributes to the development of neurodegenerative disorders. For instance, in AD, this interplay is critical for the formation of tau-containing neurofibrillary tangles, a primary hallmark of AD. Indeed, hyperphosphorylation of tau protein enhances its sumoylation in a β-amyloid (Aβ)-dependent manner. Once sumoylated, tau is less ubiquitinated and degraded, favoring tau aggregation and decreasing its solubility (Luo et al., 2014). Another neurodegenerative disorder in which the competition between ubiquitination and sumoylation is relevant for disease pathogenesis is Huntington’s disease (HD). HD is caused by the expansion of huntingtin (HTT) polyglutamine repeats. Proteolytic cleavage of mutated HTT generates a pathogenic fragment (HTTEX1P) that creates toxic aggregates in the nucleus and cytoplasm. Both ubiquitination and sumoylation target K6 and K9 of the HTTEX1P fragment and mediate opposite effects. Sumoylation increases HTTEX1P stability and aggregation, its function as transcriptional repressor and ultimately, its neurotoxicity (Steffan et al., 2004). Conversely, ubiquitination reduces HTTEX1P neurotoxicity probably by promoting its degradation (Kalchman et al., 1996). Consistent with this, in a *Drosophila* model of HD, sumoylation and ubiquitination of HTTEX1P worsen and abrogate neurodegeneration, respectively (Steffan et al., 2004).

To date, the functional interplay between ubiquitination and sumoylation in ID pathogenesis has been poorly characterized. Growing evidence suggests that a defective cross talk might underlie, at least partially, synaptic dysfunction observed in certain forms of ID. As described above, *SUMO3*, the E3 ubiquitin ligase *TTC3* and the deubiquinating enzyme *USP16* are located within the DSCR and are triplicated in DS individuals (Adorno et al., 2013; Gardiner, 2006; Saran et al., 2003). The hyperabundance of *SUMO3* could either decrease the ubiquitination of those substrates for which ubiquitination and sumoylation compete or enhance the ubiquitination of those targets for which the two PTMs operate in synergy. Triplication of *TTC3* may also lead to an unbalanced ubiquitination/sumoylation that selectively involves TTC3 substrates. For instance, AKT, a major target of TTC3, is also a known SUMO target, raising the possibility that enhanced AKT ubiquitination impairs its sumoylation status in DS patients. Whether other TTC3 substrates are subjected to the same regulatory mechanism is not known as the majority of TTC3 targets are not identified yet. Thus, besides assessing the level of AKT sumoylation in DS, it is of great interest to identify novel TTC3 substrates and investigate whether they are also targeted by sumoylation. A second ID form in which unbalanced ubiquitination/sumoylation could potentially play a central role is FXS. As mentioned above, novel pathologic missense point mutations in the *FRM1* gene are closely located to the SUMO site K130 and likely interfere with FMRP sumoylation (Khayachi et al., 2018; Prieto et al., 2020). Furthermore, FMRP is also subjected to activity-dependent ubiquitination by the APC/C complex (Hou et al., 2006; Huang et al., 2015). If ubiquitin and SUMO machineries competed for the same K residues in FMRP sequence, defective sumoylation may promote its ubiquitination. Alternatively, if sumoylation, which is required to release FMRP from dendritic mRNA granules (Khayachi et al., 2018), is instrumental to subsequent ubiquitination, these mutations may affect FMRP degradation, resulting in reduced local translation. Finally, it is also tempting to consider this possibility in regard to the AS. Indeed, ARC, a major synaptic substrate of UBE3A, is also subjected to sumoylation (Nair et al., 2017). Together, the involvement of an unbalanced cross talk between ubiquitination and sumoylation in synaptic dysfunction of ID is an exciting hypothesis and, if confirmed by further experimental data, it would define novel principles underlying the molecular logistics of synapse physiology and the pathogenesis of a spectrum of neurodevelopmental and psychiatric disorders.

## Conclusions and Perspectives

As summarized in this review, a wealth of studies clearly demonstrated pivotal roles for ubiquitination and UBLs in the assembly and refinement of neuronal circuits, maintenance of neuronal homeostasis and the emergence of complex cognitive functions. The fine spatiotemporal regulation of these PTMs is of primary importance during brain formation, a process that is characterized by intense synaptic plasticity and, on the other end, by high susceptibility to toxic insults. Therefore, genetic or environmental challenges to ubiquitination and sumoylation pathways critically contribute to the etiology of synaptopathies, including ID. Concerning neddylation, although it might be involved in ID, no evidence is currently available to support this hypothesis. The molecular mechanisms that underpin impaired ubiquitination and sumoylation in ID are unclear as well as a comprehensive view of neuronal ubiquitome and sumoylome is lacking. Here, we provide a few outlooks that are crucial to better understand the role of these two regulatory systems in the brain.

A major limitation to advance our knowledge on the pathophysiological role of ubiquitination and UBLs concerns the technical challenges to detect endogenous substrates. These modifications are transient and restricted to specific subcellular compartments, developmental stages, and states of neuronal activity. This results in scarcely abundant steady-state levels of modified targets. In recent years, new MS-based strategies were developed to efficiently capture ubiquitinated, sumoylated, and neddylated proteins in native tissues. For example, ubiquitomes and sumoylomes may be enriched using a novel monoclonal antibody that recognizes a di-glycine tag on K residues of trypsinized peptides that is present on ubiquitinated and sumoylated proteins solely (Na et al., 2012; Tammsalu et al., 2015). This technique was recently combined with CRISPR-Cas9 genome editing of *NEDD8* gene to reveal the neddylome in HEK cells (Vogl et al., 2020). Alternatively, the *in vivo* proximity-dependent biotin identification (iBioID) approach may efficiently detect substrates of specific ubiquitin and UBL machineries (Coyaud et al., 2015; Pirone et al., 2017).

A fertile area of future research will be the investigation of environmental risk factors for ID that might affect neuronal ubiquitination and UBLs. As previously mentioned, ID-linked environmental factors comprise alcohol and drug abuse and infections during pregnancy, birth complication and severe malnutrition. For instance, prenatal exposure to one of the most commonly used anticonvulsant drugs, the valproic acid, is associated with an increased risk of autism (Christensen et al., 2013) and a few reports suggest that it may regulate the UPS (Kwon et al., 2013). Microarray transcript and proteome profiling of mouse models of fetal alcohol syndrome disorders (FASD) revealed a significant downregulation of the proteasomal function (Green et al., 2007; Mason et al., 2012). In particular, the E2 conjugating enzyme UBE2N, which is known to be critical for neurodevelopment (Muralidhar and Thomas, 1993), is among the identified proteins. Similarly, fetal cortices exposed to alcohol display an increased sumoylation of the Heat shock protein 1 (HSP1), a fundamental cellular sensor of environmental proteotoxic stress, resulting in prolonged activation of HSP1 (El Fatimy et al., 2014). The E3 SUMO ligase PIASy is upregulated in alcohol-treated cultured cells and drives the induction of autophagy (Ran et al., 2018), a well-known mechanism involved in synapse assembly and maturation (Tomoda et al., 2020). Yet, whether alcohol-dependent PIASy upregulation could contribute to synaptic dysfunction in FASD is not known. Perinatal asphyxia (PA) is an obstetric complication derived from impaired gas exchange that inevitably leads to aberrant synaptic networks and severe clinical manifestations, such as epilepsy, schizophrenia, and ID. A number of studies reported enhanced ubiquitination levels in striatal and hippocampal synapses of PA animal models (Capani et al., 2009; Saraceno et al., 2012). Furthermore, hypoxia triggers sumoylation of Na_V_1.2 channels (Plant et al., 2016), which provides the earliest neuronal response to hypoxia and is a major determinant of hypoxia-induced neuronal death (Weber and Taylor, 1994). Since TTX-induced inhibition of sodium currents prevents neuronal death in acute hypoxia (Taylor et al., 1995), modulation of Na_V_1.2 sumoylation might represent a novel target to develop neuroprotective therapies (Plant et al., 2016). Finally, nicotine administration at concentrations achieved by smokers inhibits the UPS in the prefrontal cortex of adult mice and influences synaptic plasticity by regulating the levels of multiple PSD proteins, including scaffolding molecules and receptors (Rezvani et al., 2007, 2012). However, the direct effect of smoking during pregnancy on the synaptic ubiquitome of fetal brains remains elusive. Altogether, these examples support the hypothesis that ID-linked environmental risk factors affect ubiquitin and UBLs pathways, which in turn may contribute to ID pathogenesis. As these factors typically alter multiple cellular pathways, it would be of great interest to dissect the weight of defective ubiquitination and UBLs to ID onset.

At present, no effective medical therapies are available to treat ID. Defining the roles of ubiquitination and UBL modifications and the molecular mechanisms underlying these pathways in physiological and pathologic conditions could open new venues to identify novel therapeutic strategies. Given that ubiquitination and UBLs virtually regulate all cellular processes, they are attractive drug targets. For example, compounds that modulate ubiquitination, sumoylation, and neddylation have been tested to treat cancer and other pathologies (Landré et al., 2014; Nawrocki et al., 2012; Bernstock et al., 2018). However, the majority of the molecules that are currently being tested activates or inhibits global cellular ubiquitination or UBL modifications, thus inevitably leading to relevant side effects. Therefore, the development of new compounds that precisely modulate individual components or branches of ubiquitin and UBL systems and their interaction with specific substrates is urgently needed. Finally, a better elucidation of synaptic ubiquitination and UBL pathways will be of invaluable help to move toward precision medicine.

## Synthesis

Reviewing Editor: Juan Burrone, King’s College London Decisions are customarily a result of the Reviewing Editor and the peer reviewers coming together and discussing their recommendations until a consensus is reached. When revisions are invited, a fact-based synthesis statement explaining their decision and outlining what is needed to prepare a revision will be listed below. The following reviewer(s) agreed to reveal their identity: NONE.

The review provides a comprehensive description of both Ubiquitination and Sumoylation in the process of synapse function and how alterations in these pathways may be linked to intellectual disabilities. Although many reviews exist that discuss Ubiquitination and Sumoylation independently, the novelty here is that both are discussed in the same review and evidence for crosstalk between the two pathways is presented. The link to disease is timely. Overall, the review is well written and will provide an important source of information on the role of post-translational modifications on synaptic physiology and pathophysiology. However, there are a few aspects of the review that could be improved before publication. Below are comments that the authors should consider as suggestions to help improve the manuscript.

MAIN CONCEPTUAL COMMENTS AND SUGGESTIONS

1. In the view of this reviewer the broadness and robustness of the manuscript can be further improved by adding in the first “physiological” section a small sub-chapter describing the role of the Ubiquitin-like protein (UBL) Nedd8 (neural precursor cell-expressed developmentally downregulated gene 8) in neuronal and synaptic function. Nedd8 is the evolutionary closest UBL to ubiquitin and several relevant papers have recently shown the importance of Neddylation in synaptic function. Nedd8 binds to and allows the activity of the Ubiquitin Cullin-RING E3 ligase complexes. The different 7 Cullins control hundreds of targets, many of them of synaptic localization and in fact Cullin themselves have also been mechanistically involved in different neurodevelopmental disorders including intellectual disabilities. But beyond Culllins, and similarly to Ubiquitin and SUMO1-3, Nedd8 also directly targets hundreds of specific proteins (see Vogl et al. 2020, Nat Struct Mol Biol). In recent years, several papers have been published and deposited in electronic archives describing the involvement of Neddylation in the maturation, maintenance and plasticity of the synaptic compartment (Vogl et al. 2015 Nat Neurosci, Scudder et al. 2015 Mol Cell Neurosci., Bayraktar et al 2019 (BioRx), Brockmann et al 2019, Sci Reports, Vogl et al., Nat Struct Mol Biol 2020).

This topic has never been reviewed before and will therefore add novelty, broadness and impact to the article, though the title might probably need to slightly change The final chapter discussing target commonalities and Ub-Sumo molecular cross-talks is interesting too and Neddylation has also direct interactions with the ubiquitin system for instance via the Cullins system.

2. On the other hand, the first (functional) part of the manuscript is some times too focused on describing enzymes and targets, becoming a bit repetitive and becoming closer to a catalog than a conceptual piece. A comprehensive list of mediators and targets is indeed valuable but I think that section of the article would be benefited with additional information and discussion either of phenotypic (e.g. deep discussion of behavioral phenotypes accompanying the synaptic findings) or technical (e.g. recent controversies on the actual existence of SUMO1-conjugation) nature. Regarding this last point, I am particularly surprise with the absence of the papers of Nils Brose Lab which has been challenging with very accurate and extremely well-controlled studies the notion of SUMO1-conjugation to synaptic proteins using even the only tagged-SUMO knock-in mouse model available so far. Particularly the paper of Tirard et al PNAS 2011 (coauthored by Frauke Melchiord) and a subsequent eLife 2017 paper by Daniel et al, as well as another open discussion by Daniel et al in Front. Cellular Neurosci. are excellent materials to bring very relevant discussions (about unspecific antibodies, affinity purification troubles, synaptic localizations, etc.) that have not been properly addressed so far in the field of Ub and UBLs. I do not want to suggest with these comments that SUMOylation does not exist in synapses; there is substantial evidence that strongly suggests it, as the presence of the enzymatic SUMOylation apparatus in the synaptic compartment. But perhaps not all versions of SUMO have the same presence at distal neural sites or this only occurs under certain stimulation conditions, this kind of arguments could also be examined.

Our scientific community is acknowledging that we are facing a reproducibility problem and consequently about the importance of publishing papers with negative results, mainly if they are very very-controlled. This is great opportunity to follow and promote that path. In case the Editor considers that the final length might be an issue, I would recommend shortening the long description of targets to prioritize more conceptual discussions.

3. Finally, the section about ID is much more fluent and eclectic even though the pathophysiological mechanisms of these disorder are rather unclear yet. This section is an excellent counter side to balance physiology and disease. The two main syndromes chosen are thoroughly described with all the mechanistic insight available in the literature. In my opinion, the authors’ decision to leave out of the description, other syndromes (e.g. the ones listed in Table I or Prader-Willy or Kaufman Oculocerebral Syndromes) and enzymes responsible for ID disorders, although it may be justified for reasons of space, also makes this section lose some deepness and perspective. Many of these cases are actually listed in Table I, which is not even discussed. Taking this into account, I would suggest adding a small section after the Down Syndrome description to discuss at least in a general and conceptual rather than descriptive manner, for example, an analysis could be carried out according to groups of enzymes, classes of E3 ligases, types of potential targets, validations with rodent models, clinical typology that distinguishes between disorders with essentially functional pathology vs. anatomical comorbidities, etc.

MINOR COMMENTS

4. In lane 3-5 the author/s claim “Among PTMs, ubiquitination and sumoylation share several features, such as the structure of the small protein modifiers (ubiquitin and Small Ubiquitin-like Modifier, SUMO), the enzymatic cascades mediating the conjugation process and the targeted aminoacidic residue.”

The UBLs are grouped in a large family of proteins that include SUMO but also Nedd8, Fub1, Isg15, Fat10, Hub1, Ufm1, Urm1 and the autophagy-related proteins Atg8 and Atg12. In fact the family does not include ubiquitin itself. This sentence should be corrected accordingly. In addition, in the present form the authors could involuntarily and in a tacit way, favor the interpretation of three erroneous concepts. 1. That ubiquitin is part of the UBLs family. 2. That Sumo is the only UBL. 3. That there are no UBLs with relevance in the central nervous system and synaptic physiology.

To avoid this potential misunderstanding, the sentence should be modified. Furthermore it would be desirable that the authors provide the complete list of UBLs.

5. In line 49: “...that can interfere with neuronal pathways...”

Do the authors mean neuronal intracellular, signaling pathways? It´s a bit misleading.

6. In line 58: “PTMs refer to covalent but reversible enzymatic modifications of target proteins, following their translation (Bürkle, 2001). They typically consist in the addition of a functional moiety, which can be either a chemical group or a small peptide, to specific residues of target proteins.”

This statement is right in principle though it also sounds incomplete in different aspects. The PTMs might also be irreversible like in the case of proteolysis (there are several families of highly relevant proteases, particularly relevant in this review describing several DUBs) and amino acid modifications (e.g. deamination). To be accurate, these two other variants of PTMs might be mentioned. In addition, the not only chemical groups or peptides can be bound to proteins but also complex molecules (e.g. glycosylation, AMPylatoin, ADP ribosylation, etc). It might be desirable that the authors modify the paragraph accordingly.

7. In line 62: “In neurons, PTMs have been extensively investigated and modulate virtually all pathways that are required to ensure proper synaptic transmission, such as presynaptic NT release (Hegde and DiAntonio, 2002; Schorova and Martin, 2016; Takahashi et al., 2003) and trafficking of NT receptors (Diering and Huganir, 2018; Luscher et al., 2011; 65 Schorova and Martin, 2016; Widagdo et al., 2017).

This paragraph attempts to be an exemplified introduction of the multiple actions that PTMs can exert in synapses. I agree with the authors that it is an important sentence. As such it might be desirable to include the description of a broader list of examples of the actions executed by all PTMs on the many different aspects of the synaptic physiology.

8. In line 69: “In this review, we focus on the role of ubiquitination and sumoylation in synapse physiology and their implication in the molecular pathogenesis of intellectual disability (ID), a generalized neurodevelopmental disorder that manifests in a wide range of brain pathologies (Aicardi, 1998).”

Considering that ID would be a central topic along the review I would suggest the authors to provide additional and more recent references.

9. In line 77: “E1 ubiquitin enzymes bind to and activate free ubiquitin.”

I would suggest to very briefly explain what “Ub activation” means.

10. In line 82: “The human genome encodes two E1, ∼50 E2 and ∼600 E3 82 enzymes. Thus, substrate specificity mainly relies on different combinations of E2-E3 proteins.”

This is partially correct because in some cases the target specificity is mainly given by a large set of substrate receptor and adaptors. In fact this is the case of CRLs E3 ligases, which targets the largest amount of proteins in the Ub system. This should be clarified.

11. In line 137 “As this phenotype is partially rescued by the inhibition of either the proteasome or the E3 ligase Siah1, “

-It might be more accurate to rephrase it as “ As this phenotype is partially rescued by either the inhibition the proteasome or the downregulation E3 ligase Siah1,...”

12. In line 234 “ Eventually, proteomic approaches aimed at identifying synaptic ubiquitome in rat brains revealed that the Ca2+/Calmodulin-dependent kinase II (CaMKII), which is crucial for the expression of synaptic plasticity (Coultrap and Bayer, 2012), is ubiquitinated (Fig. 1A).”

-What is the citation describing this?

13. In line 239: “Ubiquitination was also studied at inhibitory synapses (Fig. 1C). The number of surface GABAARs is critically modulated by the ubiquitin-like protein Plic-1 (also named ubiquilin)...”

- Ubiquilin, is actually an ubiquitin-associated chaperon and the term ubiquitin-like protein (originally employed in the rather old referred paper) nowadays is used to name UBLs and therefore it would be misleading. To avoid misunderstanding of non-expert readers, the term UBL should be reserved to describe the UBL family members.

14. In line 278: “For example, neuronal overexpression of SUMO1 in vivo affects the density of dendritic spines and worsens behavioral performances in learning and memory tests (Matsuzaki et al., 2015).”

- It is somehow surprising that the authors choice to start the description of the role of SUMO in synapses with this paper. The overexpression of ubiquitin and UBLs is an ambiguous, misinforming and thus outdated approach that should be used and reserved for very special experimental cases. The lost of endogenous stoichiometry upon SUMO (or other Ub/UBLs) overexpression is known to potentially give raise to artefactual targeting and downstream effects. The paper will be mentioned again later on in the review. If the authors are still interested in mentioning this paper should at least express concerns about this well-known technical drawbacks.

15. In line 356: “A primary presynaptic function controlled by the SUMO pathway is SV docking/priming through sumoylation of RIM1α and synapsin Ia (SynIa) (Fig. 1A). Sumoylation of SynIa triggers its association with SVs and facilitates SV anchoring at the presynaptic membrane (Tang et al., 2015).”

- I already mentioned the papers of Daniel et al above (eLife and Frontiers Cell Neurosci). In those papers, the authors specifically test in different ways the SUMO1 conjugation to several of the best-described synaptic proteins of the literature including RIM1 and Synla. They could not see the described conjugation. In line 410 something similar happen for the case of GluK2. These cases should be discussed in the light of the Daniel´s findings too.

16. In line 376: “However, mGlu7R is the sole member shown to be sumoylated in vivo”.

The in vivo concept often has different interpretation in the different fields. Meanwhile cell cultures models can be considered “in vivo” by many biochemists accustom to work with recombinant proteins and assays, the same approach is rated as “in vitro” for many neuroscientists. To avoid misunderstandings, it would be ideal to clarify the experimental model employed.

17. In line 767: “For example, compounds that modulate sumoylation have been tested to treat cancer and ischemia (Bernstock et al., 2018).”

I would add the word ubiquitination “For example, compounds that modulate ubiquitination and sumoylation have been tested to treat cancer and ischemia (Bernstock et al., 2018).”, adding of course an adequate citation.

18. A recent remarkable paper in Plos Biol. by Feng et al. (2019) “The deubiquitinase USP6 affects memory and synaptic plasticity through modulating NMDA receptor stability” describes the effects of Usp6, a hominoid deubiquitinating enzyme previously implicated in intellectual disability and Asperger Syndrome. This is a interesting paper involving the characterization of the humanized mouse models expressing Usp6 in forebrain pyramidal neurons, a kind of approach that is substantially different to the studies described in the paper which are sometimes repetitive in their experimental nature. A comment on this paper could be included either associated to the role of ubiquitination and NMDA function and/or associated to intellectual disabilities.
